# Thermo‐Chemically Modified Silk Scaffolds Reveal Niche‐Driven Regulation of Hematopoiesis and Fibrosis

**DOI:** 10.1002/smll.202513071

**Published:** 2026-01-30

**Authors:** Christian A. Di Buduo, Carolina P. Miguel, Giulia Della Rosa, Vittorio Abbonante, Santo Diprima, Delfina Tosi, Marta Filibian, Daniele Cattaneo, Jugal Kishore Sahoo, Nicola Tirelli, Alessandra Iurlo, Umberto Gianelli, David L. Kaplan, Alessandra Balduini

**Affiliations:** ^1^ Department of Molecular Medicine University of Pavia Pavia Italy; ^2^ Polymers and Biomaterials Lab Istituto Italiano Di Tecnologia Genova Italy; ^3^ Center for Omics Sciences IRCCS San Raffaele Scientific Institute Milan Italy; ^4^ Department of Health Sciences University of Milan Unit of Pathology ASST Santi Paolo e Carlo Milan Italy; ^5^ Centro Grandi Strumenti University of Pavia Pavia Italy; ^6^ Hematology Division Foundation IRCCS Ca’ Granda Ospedale Maggiore Policlinico Milan Italy; ^7^ Department of Oncology and Hemato‐Oncology University of Milan Milan Italy; ^8^ Department of Biomedical Engineering Tufts University Medford Massachusetts USA

**Keywords:** bone marrow, fibrosis, megakaryocytes, mesenchymal stem cells, platelets, silk fibroin, transforming growth factor beta1

## Abstract

Recreating the human bone marrow microenvironment in vitro remains a critical challenge in advancing our understanding of hematopoiesis and its disruption in disease. Here, we present a fully tunable bone marrow model based on silk fibroin scaffolds engineered through thermo‐chemical processing to replicate the mechanical and structural features of native marrow. This 3D platform integrates mesenchymal stromal cells (MSCs) and supports the functional differentiation of hematopoietic stem and progenitor cells (HSPCs) into mature megakaryocytes and platelets. RNA sequencing of MSCs cultured on physiologically tuned scaffolds revealed transcriptional programs closely aligned with native stroma, validating the fidelity of the engineered niche. The model captures essential marrow dynamics, including matrix remodeling and perfusion flow, enabling direct assessment of thrombopoietic function. To simulate fibrotic remodeling, scaffolds were functionalized with TGF‐β1, inducing MSC transition into myofibroblast‐like cells and recreating pathological features of myeloproliferative neoplasms. In this context, patient‐derived HSPCs exhibited impaired megakaryocyte maturation and aberrant calcium signaling, partially restored by interfering with calcium flux. To quantify microenvironment‐driven dysfunction, we calculated changes in megakaryocyte size distribution using a Divergence Index. Combined with the engineered niche, this functional metric offers a powerful and quantifiable platform to dissect dysregulated hematopoiesis and evaluate therapeutic strategies in patient‐derived systems.

## Graphical abstract

We introduce a versatile 3D platform that recreates key physical and biological features of the human bone marrow. By integrating tunable silk biomaterials, stromal cells, and human hematopoietic progenitors, the model captures both healthy and diseased microenvironments, analysis of blood formation, and its disruption in pathological conditions.

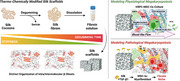

## 1 Introduction

Developing physiologically relevant in vitro models requires reproducing the structural, mechanical, and biochemical characteristics unique to each human organ. While conventional tissue culture systems have advanced basic cell biology, they fall short in capturing the spatial organization and mechanical dynamics critical to disease processes in complex tissues. Among these, the bone marrow is especially challenging to model due to its highly specialized architecture and finely regulated cellular cross‐talk that governs hematopoiesis [1, 2, 3].

Bone marrow homeostasis depends on dynamic interactions between hematopoietic stem and progenitor cells, mesenchymal stromal cells (MSCs), and a niche‐specific extracellular matrix (ECM), which together orchestrate lineage specification and differentiation [4, 5, 6, 7, 8, 9]. However, the inaccessibility of the marrow and the absence of organ‐specific platforms limit mechanistic studies and therapeutic testing. Accurately modeling this environment requires engineered systems that reproduce the marrow's mechanical compliance, molecular composition, and spatial organization. Recent efforts to replicate the bone marrow niche have included hydrogel‐based systems, decellularized matrices, and organ‐on‐chip devices [10, 11, 12, 13]. While these approaches have provided insight into niche architecture and selective lineage outputs, they often lack tunable mechanical properties or fail to support long‐term stromal‐hematopoietic interactions.

Silk fibroin, a protein biopolymer derived from *Bombyx mori* cocoons, features a repetitive Gly‐Ala‐Gly‐Ala‐Gly‐Ser sequence that drives hierarchical self‐assembly into β‐sheet–rich structures [14, 15, 16, 17]. These properties make it an ideal, tunable material for engineering tissue‐specific microenvironments [18, 19, 20]. Of primary relevance, the molecular weight of fibroin influences solution viscosity, scaffold porosity, and mechanical stiffness: higher molecular weight results in slower diffusion and greater mechanical integrity. At the same time, β‐sheet crystallinity, modulated through thermal processing, dictates degradation kinetics and structural stability [19, 21].

By tailoring these features, we engineered silk‐based scaffolds that support marrow‐like mechanical and molecular environments. This platform enables functional modeling of hematopoietic niches, including physiological and fibrotic states, and supports the integration of patient‐derived cells to study disease‐specific mechanisms and therapeutic responses. Our approach represents a generational shift from conventional soluble‐factor–based systems to engineered microenvironments with biophysical control, revealing mechanisms of bone marrow function previously inaccessible in standard models.

## 2 Results

### 2.1 Decoding the Architecture of the Bone Marrow Niche Using Micro‐CT and Advanced Imaging

**FIGURE 1:**
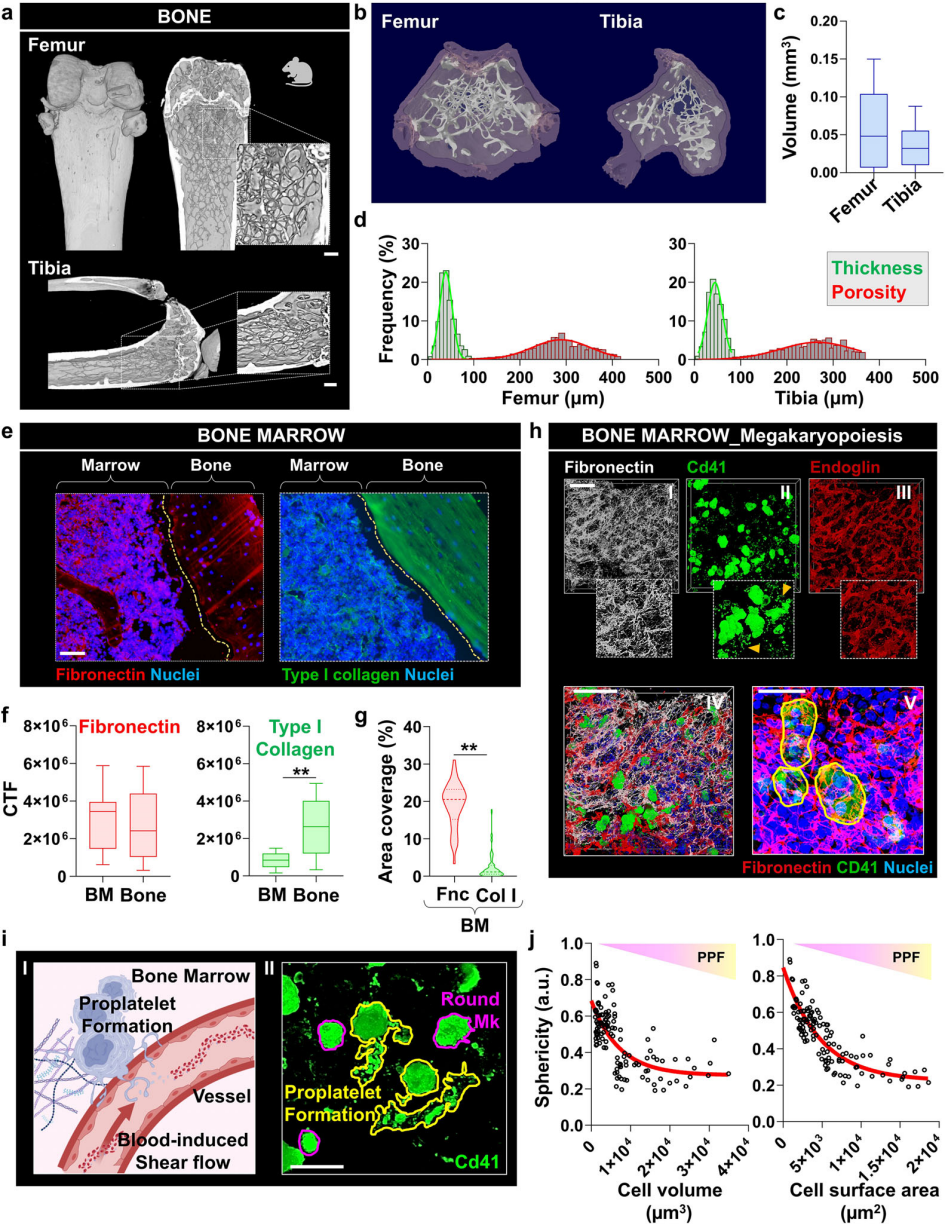
Micro‐CT and immunofluorescence imaging of bone and bone marrow. (a) Micro‐CT imaging of femur and tibia, showing structural differences and highlighting trabecular bone architecture. The insets provide zoomed‐in views of trabecular networks (scale bar = 400 µm). (b) Cross‐sectional 3D reconstructions of the femur and tibia, illustrating volumetric and structural features. (c) Quantitative comparison of bone volume between the femur and the tibia, presented as box‐and‐whisker plots (median, interquartile range, and min–max). (d) Frequency distribution histograms showing thickness (green) and porosity (red) of trabeculae in the femur and tibia. (e) Immunofluorescence staining showing the spatial organization of Fibronectin (red) and Type I collagen (green), in the bone marrow compartment and in the compact bone (scale bar = 100 µm). (f) Quantitative analysis of Fibronectin and Type I collagen in the bone marrow (BM) and the bones. Box plots indicate significant differences in expression levels between bone marrow and compact bone (CTF = corrected total fluorescence; data are presented as box‐and‐whisker plots (median, interquartile range, and min–max). n = 50 per group. Statistical significance was determined using an unpaired two‐tailed Student's t‐test. **p < 0.01). (g) Comparison of the area coverage of Fibronectin (Fnc) and Type I collagen (Col I) in the bone marrow. (Data are presented as violin plots. n = 80 per group. Statistical significance was determined using an unpaired two‐tailed Student's t‐test. **p < 0.01). (h—I‐IV) Bone marrow megakaryopoiesis visualized via immunofluorescence staining for fibronectin, Cd41, and Endoglin. Panels show distinct localization of proplatelet‐forming megakaryocytes (yellow arrows) within vessels (scale bar = 100 µm). (h—V) The inset highlights megakaryocytes (green) embedded in a Fibronectin network (red) (scale bar = 30 µm). (i—I) The schematic representation illustrates proplatelet formation (PPF) and platelet release within the bone marrow microenvironment. (i—II) Immunofluorescence imaging of Cd41^+^ megakaryocytes (Mk, green) shows significant heterogeneity in volume and area coverage of cells during their transition from small round progenitors into proplatelet‐forming cells (scale bar = 50 µm). (j) Analysis of cell characteristics, including sphericity (a.u. = arbitrary unit), cell volume, and cell surface area. Plots show the relationship between cell sphericity and cell volume/surface area (n = 123).

Micro‐computed tomography (Micro‐CT) was employed to analyze the intricate architecture of the mouse femur and tibia, offering a detailed depiction of the spongy structure of trabecular long bones (Figure 1a). High‐resolution 3D reconstructions (Figure 1b) clearly illustrate the porous organization, an essential feature of the hematopoietic microenvironment. Quantitative analyses of the volume, thickness, and pore diameter of the trabeculae revealed comparable parameters between the femur and tibia (Figure 1c,d).

Immunofluorescence staining provided spatial localization of the two key ECM components of the bone and bone marrow known to regulate platelet production, Fibronectin and Type I collagen [22, 23]. Fibronectin was widely distributed throughout the bone and the marrow, emphasizing its role in maintaining the structural integrity of the whole hematopoietic niche (Figure 1e, left). Type I collagen expression was weaker in the bone marrow, while it was mostly localized at the compact bones, suggesting a prominent role in the bone structural organization (Figure 1e, right). Quantitative analysis confirmed these differences, with Fibronectin showing significantly higher area coverage in bone marrow than Type I collagen (Figure 1f,g). In this context, Endoglin, one of the major antigen markers of MSC and endothelial cells, was observed in perivascular regions and throughout the marrow, within the intravascular space, indicating active stromal support for mature blood progenitor cells approaching maturation and migration into circulation. Cd41^+^ cells, a megakaryocyte marker, were found clustered near Endoglin and embedded within Fibronectin‐rich networks (Figure 1h). The vasculature serves as a dynamic site for proplatelet formation, with elongation directed toward the vascular lumen, where shear forces facilitate platelet release into the bloodstream (Figure 1i I) [24, 25]. Our findings demonstrate that megakaryocytes exhibit substantial heterogeneity in their volume and surface area coverage during their physiological transition into proplatelet‐forming cells (Figure 1i II). This transition is characterized by an increase in surface area‐to‐volume ratio and a decrease in cellular sphericity, reflecting the dynamic morphological changes associated with proplatelet extension (Figure 1j). Smaller megakaryocytes exhibited less extensive surface area coverage, indicating an earlier stage of maturation. Overall, the integration of micro‐CT and high‐resolution imaging enabled us to identify the critical components required to construct an optimal supportive microenvironment for thrombopoiesis.

### 2.2 Engineering Silk Fibroin Structure to Model the Bone Marrow Microenvironment

**FIGURE 2:**
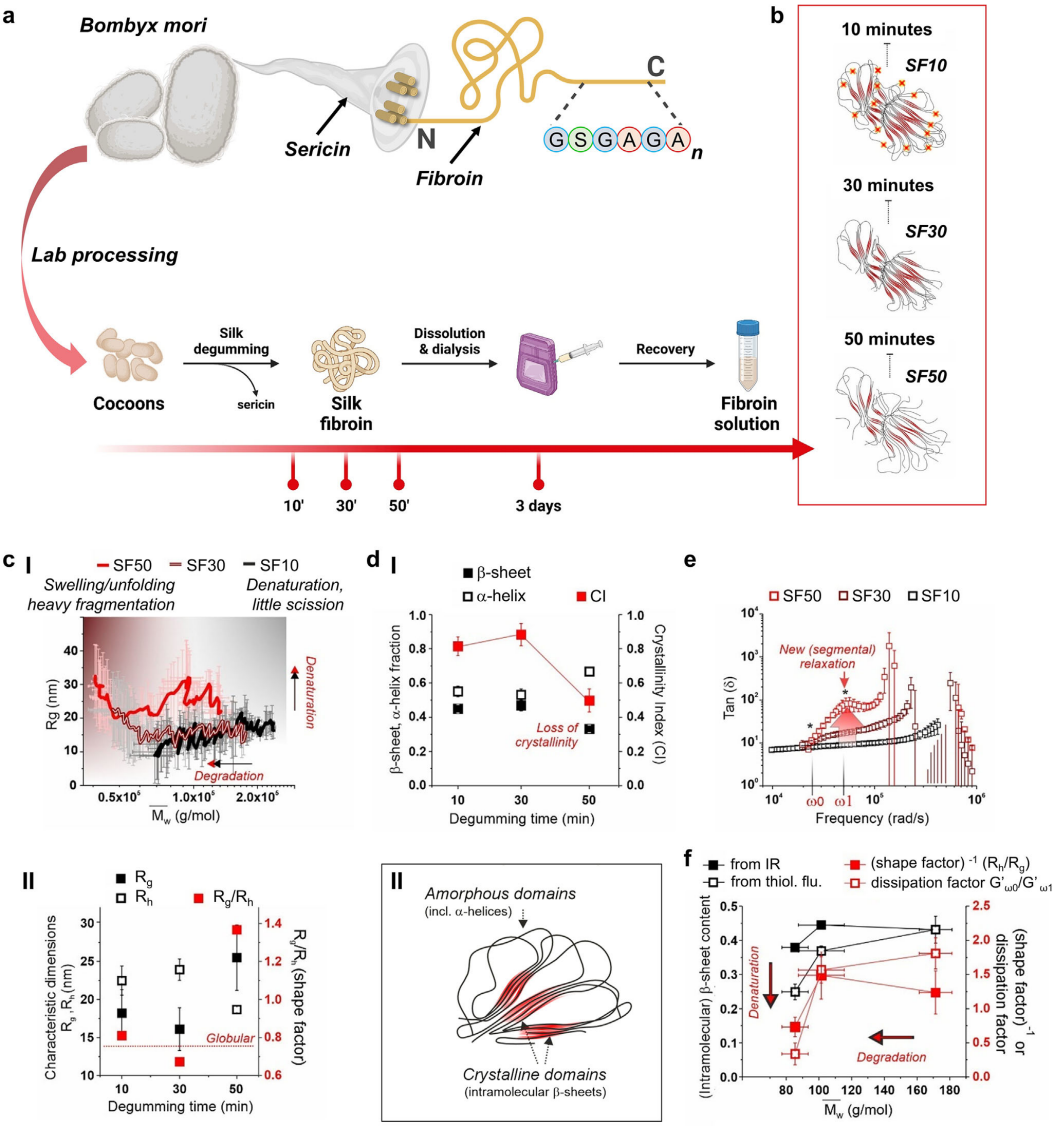
Structural and rheological characterization of silk fibroin variants. (a) Schematic overview of silk fibroin (SF) processing from *Bombyx mori* cocoons. Key steps include degumming at different timings (10, 30, 50 min) to remove sericin, dissolution and dialysis, and recovery to obtain silk fibroin solutions with varying molecular weights. (b) Structural depiction of SF10, SF30, and SF50 proteins, highlighting their secondary structure organization as determined by degumming time. Increasing degumming time correlates with increased fragmentation and reduced molecular weight. (c—I) Weight‐average molar mass ($\bar{M_{w}}$) versus radius of gyration (*R_g_*) of SF10, SF30, and SF50 obtained via asymmetric flow field‐flow fractionation technique (AF4). (c—II) Correlation between *R*_g_ and hydrodynamics radius (*R_h_*) of SF10, SF30, and SF50 (left axis) and shape factor (*R_g_*/*R_h_*). (d—I) β‐sheet and α‐helix fractions (areas of peaks at ∼ 1515 and ∼ 1545 cm^−1^ (from Gaussian fittings) divided by the total area of the amide II band) and crystallinity index (CI; from the 1515/1545 cm^−1^ absorbance ratio). Please note that here we have employed the amide II rather than the possibly more common amide I band; the former is equally informative about protein secondary structures [35], and indeed the two bands provide rather similar information [36], but the former is advantageous for fully hydrated samples like ours, because it does not suffer from interference with the water bending (δOH) absorption. (d—II) Schematic representation of silk fibroin crystalline domain. (e) tan (δ) of 0.2% % wt. SF10, SF30, and SF50 as a function of frequency (ω) obtained from diffusing wave spectroscopy (DWS). Please refer to Figure S3, for the dependency on concentration in the 0.1–4.0% range. (f) Correlation plot describing the effect of degumming‐time‐dependent degradation and denaturation process on SF10, SF30, and SF50 structural properties.

Native silk fibroin protein is formed as a dimer, composed of a heavy ∼390 kDa chain and a light ∼25 kDa chain linked by a single disulfide bond [18]. The fibroin is extracted from *B. mori* silk cocoons by degumming, followed by dissolution in Lithium Bromide (LiBr) and dialysis (Figure 2a) [21]. Depending on the degumming time, the silk fibroin protein molecular weight distribution can be tuned [26, 27, 28]. Here, we explored the degumming time‐dependent change in silk fibroin to optimize the material properties and consequent cell behavior related to bone marrow modeling. Hereinafter, silk fibroin samples degummed for 10, 30, or 50 min will be named silk fibroin (SF)10, SF30, and SF50, respectively (Figure 2b).

During the degumming process, the mass fraction lost increased with degumming time (25.2 ± 0.2% wt. after 10 min, 26.0 ± 0.2% wt. after 30, 28.4 ± 0.4% wt. after 50; black squares; Table 1). Sericin was completely removed after 10 min [29]. The mass loss was likely due to degradation because of hydrolytic cleavage events in the protein backbone in alkaline conditions. Indeed, asymmetric flow field‐flow fractionation (AF4) with MALS/RI detection showed a shift of the whole molecular weight distribution of SF10 to considerably lower values in SF30, which is consistent with previous reports of fibroin degradation under similar conditions [19, 29, 30]. The progression to SF50 determined further changes in molecular weight with respect to SF30, though this difference was not statistically significant (Figure S1a, left). This is consistent with prior reports using comparable degumming conditions showing no major differences in amino acid composition at intermediate/long degumming times [19]. The similar molecular weight, observed for SF30 and SF50 in our study, likely reflect this preserved amino acid composition. Dimensionally, SF10 and SF30 were similar, with comparable values of *R_g_*, despite their difference in molecular weight ($\bar{M_{w}})$; while *R_g_* increased for SF50 (Figure S1a, right). Taken together, these findings suggest a two‐step process: an initial phase of significant fibroin degradation, followed by structural changes driven by molecular unfolding (Figure 2c I) [31, 32, 33].

**TABLE 1:** Characterization data of SF recovered after degumming ^a)^.

	Mass loss [wt. %]	$\bar{M_{n}}$ ^b)^ [g mol^−1^]	$\bar{M_{w}}$ ^b)^ [g mol^−1^]	Ð ^c)^	*R*_g_ ^b)^ [nm]
**SF10**	25.2 ± 0.2	1.4*10^5^ ± 0.1*10^4^	1.7*10^5^ ± 0.1*10^4^	1.15 ± 0.01	18 ± 3
**SF30**	26.0 ± 0.2	8.9*10^4^ ± 1.5*10^4^	1.0*10^5^ ± 1.4*10^4^	1.18 ± 0.07	16 ± 3
**SF50**	28.4 ± 0.4	7.2*10^4^ ± 1.0*10^4^	8.4*10^4^ ± 0.8*10^4^	1.18 ± 0.06	26 ± 4

^a)^ Averages ± SD over three independent samples

^b)^ % of mass loss from the fibroin recovery after dialysis.

^c)^ The number‐ ($\bar{M_{n}}$) and weight‐average ($\bar{M_{w}}$) molar masses, their dispersity Ð ($\bar{M_{w}}/\bar{M_{n}}$) and the radius of gyration (*R_g_*), were obtained *via* asymmetric flow field flow fractionation (AF4) using refractive index and multi‐angle static light scattering detectors.

Several other experimental results confirmed this interpretation. The fibroin shape, expressed through its *R_g_*/*R_h_* ratio, hydrodynamic radius (*R_h_*), measured via dynamic light scattering (DLS), showed a shape factor of 0.65‐0.8 for SF10 and SF30, respectively, and up to 1.3 for SF50 (Figure 2c II). This indicates the first two have a similar compact structure [34], while the latter has undergone significant unfolding. In addition, the amide II band in the FTIR spectrum has components associated with β‐sheets and α‐helices (1510‐1520 and 1540–1550 cm^−1^, respectively; Figure S1b). We have employed the amide II rather than the amide I band, as the former is equally informative about protein secondary structures and provides similar information [35, 36]. Amide II is advantageous for fully hydrated samples like those in the present work because it does not suffer from interference with absorption from water bending (δOH) [35, 36, 37]. The ratio of the two components, often referred to as crystallinity index (CI) (Figure 2d I, 2d II), was similar for SF10 and SF30 but lower for SF50 (empty black squares in Figure 2d I), suggesting that the longer degumming decreased the content of the peptide domains responsible for correct folding of the fibroin into β‐sheets. Accordingly, the β‐sheet‐related fluorescence of Thioflavin‐T (ThF‐T), which is modulated by the morphology and internal order of β‐sheets [38], had a decrease in intensity from SF10 to SF50 (Figure S2), thus suggesting a major reduction in β‐sheet content with the longer degumming time. Finally, the development of segmental relaxation modes, investigated via diffusing wave spectroscopy (DWS), showed a new relaxation mode in SF50 (Figure 2e; ω1 is the peak frequency of this mode), indicating a higher molecular mobility. SF10, and to a lower extent SF30, showed a more elastic (and rigid) behavior, with lower values of tan (δ) and higher *G*′ (Figure S3). By defining a dissipation factor as the ratio of G′ outside versus within the specified region (at ω_1_), we revealed that SF50 exhibits a lower elasticity compared to both SF10 and SF30 (Figure 2f). Overall, the data demonstrated that SF10 and SF30 differ in molecular weight due to significant degradation during degumming but preserve a similar β‐sheet‐related internal order. SF50 does not undergo significantly increased degradation compared to SF30, but does differ from both SF10 and SF30 in terms of β‐sheet content.

### 2.3 3D Silk Scaffolds with Structural and Mechanical Fidelity to the Bone Marrow Microenvironment

**FIGURE 3:**
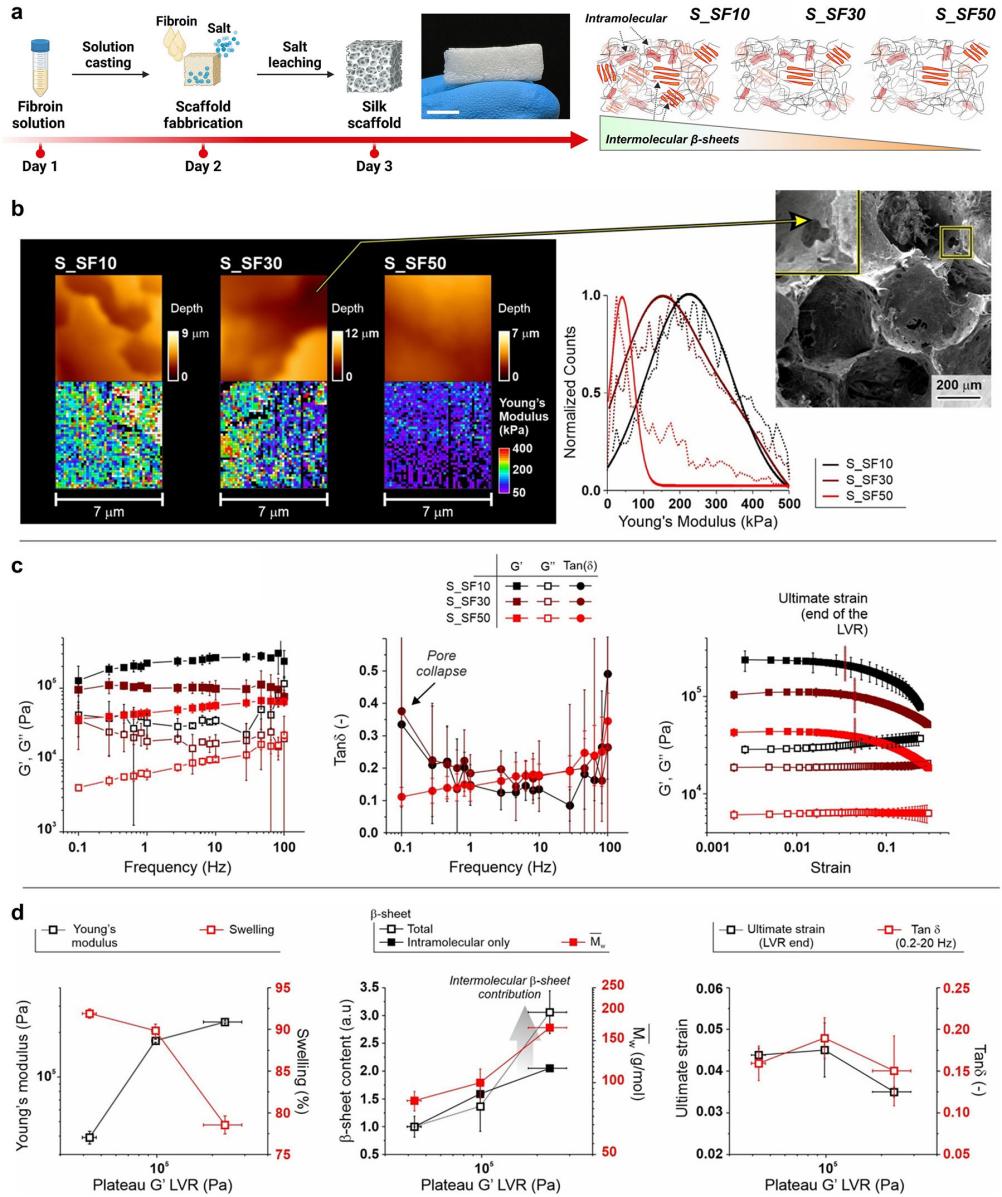
Mechanical and structural characterization of silk fibroin scaffolds. (a) Fabrication process of silk fibroin (SF) scaffolds (S_SF10, S_SF30, S_SF50) through solution casting, salt leaching, and subsequent structural stabilization (scale bar = 0.7 mm). The schematic illustrates differences in intramolecular and intermolecular *β‐sheet* contributions among the variants. (b) Left. Representative examples of height images (top) and force maps (bottom) (7.0 × 7.0 µm) acquired via AFM nanoindentation and displayed as heat maps. Center. Distribution of Young's moduli calculated from the force maps and fitted with a Gaussian model. *Right*. Representative SEM image of S_SF30 (for completeness, distribution of Young's modulus and other SEM pictures are available in Figures S4 and S5). The inset is magnified on the left of the image, and shows the presence of 1–10 µm pores, which can also be seen in the AFM height images (connecting arrow). (c) Left: Frequency dependency (0.1–100 Hz; via rotational rheology) of scaffold storage (*G*′) and loss moduli (*G*′′). C*enter*: frequency dependency of tan (δ) = *G*′′ /*G*′; please note that we ascribe the tan (δ) increase at low frequency for S_SF10 and S_SF30 to pore collapse, which is more likely in these less hydrated scaffolds. *Right*: strain dependency of *G*′ and *G*′′ (γ = 0.0021–0.3%); black and red arrows highlight the ultimate strain, i.e., the end of the linear viscoelastic region (LVR) in the *G*′ curves, identified as the γ value corresponding to a 10% decrease in *G*′. (d) Left. The Young's modulus obtained via AFM nanoindentation (black symbols) and the scaffold swelling (red symbols) scale directly and inversely with plateau *G*′. Center. The intramolecular β‐sheet content (full black symbols; from Thioflavin‐T staining of SF in solution), the total β‐sheet content (empty black symbols; from Thioflavin‐T staining of SF scaffolds), and SF weight‐average molar mass ($\bar{M_{w}}$) correlate reasonably well with the scaffold plateau *G*′. *Right*. The scaffolds’ resistance to deformation (ultimate strain, black symbols) and viscoelastic character (tan (δ) in 0.2–20 Hz; red symbols) do not show a clear correlation with the plateau (LVR) *G*′ values.

Silk fibroin scaffolds were prepared via a salt leaching method by mixing the protein solution with NaCl particles, sieved to obtain ∼500 µm diameter particles, acting as a porogen [21, 39, 40]. After drying, the scaffolds were hydrated in deionized water to solubilize the salt (Figure 3a). Hereinafter, the 3D scaffolds obtained from silk fibroin of different degumming times will be named S_SF10, S_SF30, and S_SF50, respectively (Figure 3a). Scanning electron microscopy (SEM) and atomic force microscopy (AFM) showed the three scaffolds had comparable surface roughness (RMS), with distributions in the range of 0.2–0.7 µm (Figure S4, left, and Figure S5). Conversely, Young's modulus obtained via nanoindentation (scaffold morphology, force maps and distributions in Figure 3b; force map fitted parameters presented in Table S1) showed a decrease with increasing the degumming time, in the order S_SF10> S_SF30>> S_SF50.

In rotational rheology, all samples showed elastic behavior. In the linear viscoelastic regions (LVR), storage moduli were consistently about one order of magnitude higher than loss moduli both in frequency sweeps (Figure 3c, left) and in strain sweeps (Figure 3c, right); in the former, both *G*′ and *G*′′ showed a flat dependency, confirming the elastic nature. The exception was below 1–2 Hz S_SF50's where the moduli decreased with decreasing frequency, which cannot be ascribed to the occurrence of relaxation phenomena since no corresponding peak in $\tan\left(\delta \right)$ was observed (Figure 3c, center). Amplitude sweeps showed the LVR extended similarly for all samples (Figure 3c, right).

Overall, these analyses showed that scaffold stiffness values obtained from both bulk (average G′) and surface (Young's modulus) measurements were comparable (Table 2; Figure S6). Only S_SF30 showed a statistical difference, and the two parameters appeared to scale well together (Figure 3d, left). This demonstrated a satisfactory homogeneity of the materials utilized for fabricating the bone marrow system. In parallel, a noteworthy difference was observed in the extent of water swelling: S_SF10 (79 ± 1%) was considerably lower than S_SF30 and S_SF50 (89 ± 1% and 92 ± 1%, respectively). As expected, swelling inversely correlates with stiffness, as shown by its relationship with *G*′ (Figure 3d, left). The moduli (*G*′ in Figure 3d, center) correlated well with the SF's molecular descriptors, including its molecular weight and its capacity for β‐sheet formation (both intramolecular, determined from solution measurements, and total, determined from scaffold measurements, Figures S2 and S7). Intermolecular β‐sheets (measured in scaffolds) showed a stronger correlation with silk fibroin molecular weight than intramolecular β‐sheets (measured in solution). For instance, both exhibited significant differences when moving from SF10 to SF30 and only slight differences from SF30 to SF50. This trend is reasonable, as intermolecular associations are increasingly possible with larger macromolecules. The overall mechanical characteristics ($\tan\left(\delta \right)$) of the scaffolds and their resistance to irreversible deformation (ultimate strain) were insensitive to the molecular descriptors, thus suggesting that these features depend on pore formation conditions, which were constant for all samples. The lack of interdependence between these mechanical properties and moduli was apparent in a plot of ultimate strain and $\tan\left(\delta \right)$ constant vs. *G*′ (5‐fold variation, Figure 3d, righ**t**). Thus, silk fibroin denaturation during the degumming process impacts the subsequent intermolecular β‐sheet formation during 3D scaffold production, resulting in structures having different mechanical properties. Based on the evidence that S_SF10 and S_SF50 exhibit the most significant differences, these two scaffolds were selected for downstream biological testing.

**TABLE 2:** Characterization data of SF and SF scaffolds ^a)^.

Sample (SF)	$\bar{M_{w}}$ ^b)^ [kg mol^−1^]	Scaffold	*G*′ [kPa] ^c)^		Young's modulus [kPa] ^d)^		Swelling ^e)^ [%]
			Average	Plateau	Peak	Average	
***SF10***	170 ± 10	***S_SF10***	200 ± 18	234 ± 54	230 ± 34	237 ± 12	79 ± 1
***SF30***	100 ± 14	***S_SF30***	115 ± 10	98 ± 5	170 ± 43	202 ± 12	89 ± 1
***SF50***	84 ± 8	***S_SF50***	42 ± 5	43 ± 3	37± 4	126 ± 11	92 ± 1

^a)^ All data are averages ± SD over three independent samples.

^b)^ Absolute weight‐average molar mass ($\bar{M_{w}}$) obtained via asymmetric flow field flow fractionation (AF4) and MALS detection.

^c)^ *G*′ from rotational rheology. The values were obtained *via* 5‐min averages (0.02 strain, 1.0 Hz frequency) on pre‐formed but not mechanically conditioned (see examples of such curves in Figure S6), or by averaging the LVR values during amplitude sweep tests (strain range of 0.002 – 0.03% at a frequency of 1.0 Hz).

^d)^ From AFM nanoindentation (Hertzian model) on a minimum of three 10*10 µm areas; the overall distribution was fitted with a Gaussian model of the type $y=y_{0}+\frac{2A}{\pi }\;\frac{w}{4{\left(x-x_{c}\right)}^{2}+w^{2}}$, presenting the peak (most probable) and the number average value.

^e)^ Calculated as $Swelling\;\left(\%\right)=\frac{W_{h}-\;W_{d}}{W_{d}}*100$, where *W_h_* and *W_d_* were the weight of samples, respectively, after salt washing (at the end of the preparative phase) and after freeze drying.

### 2.4 Silk Scaffold Compliance Modulates MSC Function and Thrombopoiesis

**FIGURE 4:**
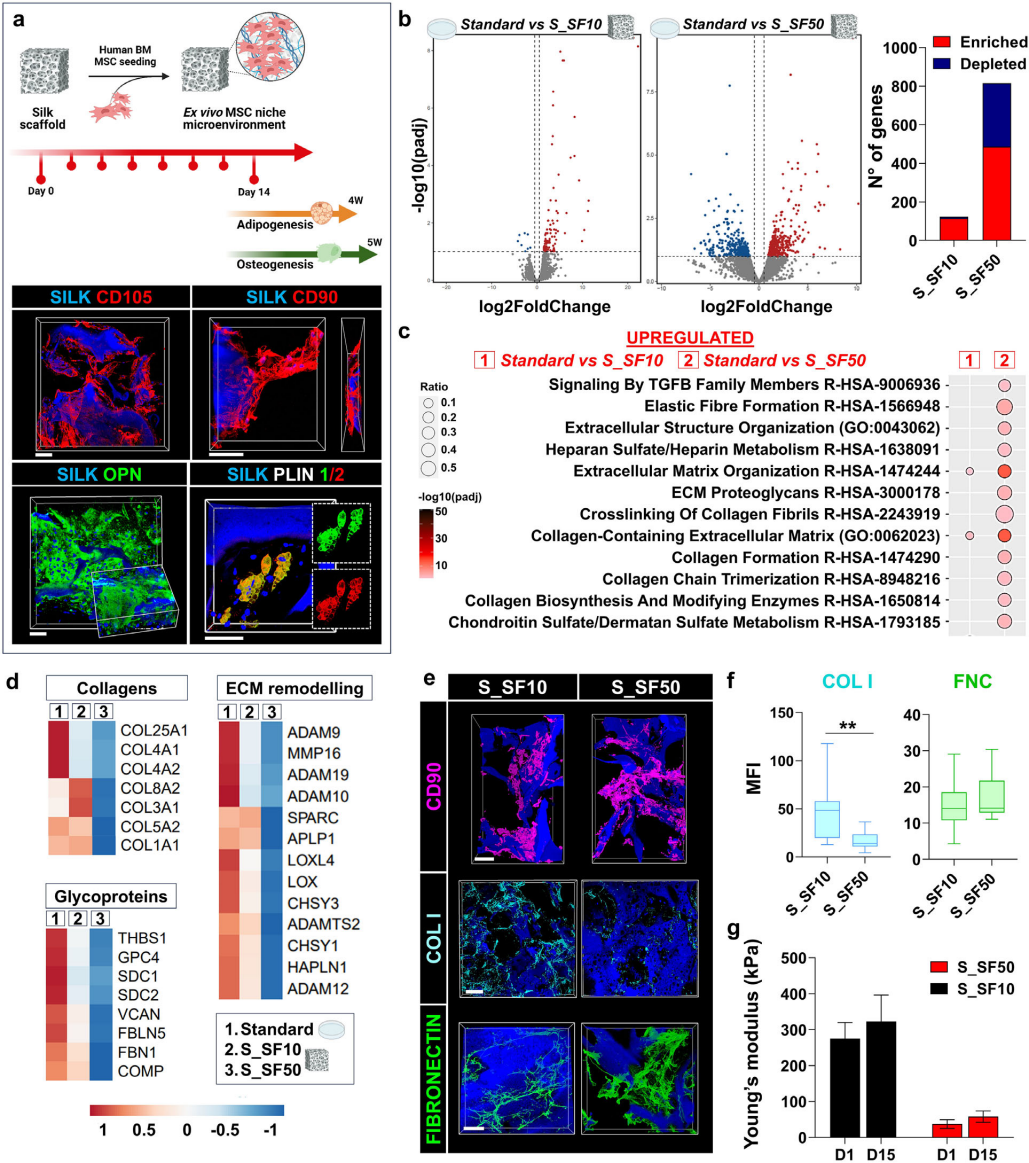
Functional assessment of silk scaffolds in ex vivo bone marrow niche models. (a) Schematic of the experimental workflow for seeding human bone marrow‐derived mesenchymal stem cells (MSCs) into silk scaffolds. MSCs were cultured for 14 days, followed by differentiation into adipogenic (4 weeks) and osteogenic (5 weeks) lineages. Immunofluorescence staining of CD105, CD90, OSTEOPONTIN (OPN), and PERILIPIN (PLIN) 1/2 indicates cellular phenotypes at different stages (scale bar = 100 µm). (b) Volcano plots comparing the gene expression profiles of MSCs cultured in standard liquid cultures (petri dish) versus S_SF10 or S_SF50 scaffolds. Analyses display differentially expressed genes, with significantly enriched (red) and depleted (blue) genes. (c) Pathway enrichment analysis of RNA‐seq data showing biological processes and pathways altered by scaffold composition. Key pathways, including extracellular matrix (ECM) organization and collagen metabolism, are shown. Circle size represents the ratio of enrichment, while color intensity corresponds to significance. (d) Heatmaps of differentially expressed genes involved in collagen synthesis, ECM remodeling, and glycoproteins, comparing standard liquid cultures, S_SF10, and S_SF50 conditions. Gene expression patterns reflect scaffold‐dependent modulation of ECM components and matrix remodeling enzymes. (e) Immunofluorescence images of MSCs in S_SF10 or S_SF50 scaffolds, stained for CD90, TYPE I COLLAGEN (COL I), and FIBRONECTIN (FNC) (scale bar = 100 µm). (f) Quantitative analysis of mean fluorescence intensity (MFI) of COL I and FNC in S_SF10 or S_SF50 scaffolds (data are presented as box‐and‐whisker plots (median, interquartile range, and min–max). n = 50 per group. Statistical significance was determined using an unpaired two‐tailed Student's t‐test. **p < 0.01). (g) Comparison of Young's modulus (mechanical stiffness) of S_SF10 and S_SF50 scaffolds on days 1 and 15 of culture (data are presented as mean ± S.D., n = 3).

A growing body of evidence demonstrates the importance of substrate stiffness and elasticity in regulating stem cell behavior, influencing crucial processes like adhesion, cytoskeletal organization, and differentiation [12, 41, 42, 43, 44]. To develop functional 3D bone marrow models, it is essential to consider the dynamic interactions between human MSCs and the biochemical and mechanical properties of the scaffold material, which directly influence MSC functions ex vivo. Specifically, characteristics such as surface microgeometry and mechanical compliance are known to significantly impact MSC bioactivity. In this study, aiming to generate a microenvironment that resembles features of the human bone marrow for supporting thrombopoiesis, we first assessed the ability of the various 3D scaffolds to maintain MSC stemness, using standard culture as a control for comparison. In the basal expansion medium, MSCs thoroughly populated the scaffolds, maintaining the expression of stemness markers, thus demonstrating the preservation of self‐renewal potential in the 3D microenvironment (Figure 4a). MSCs, with their multipotent nature, possess the remarkable ability to differentiate into various cell types. When directed toward a particular lineage, MSCs undergo significant morphological and functional modifications. We assessed MSC ‘fitness’ by differentiating them in osteogenic or adipogenic media, both in standard liquid culture and 3D scaffolds (Figure 4a). Under osteogenic conditions, MSCs differentiated into OSTEOPONTIN^+^ osteoblasts with significantly increased expression of *RUNX2, SPP1*, and *ALPL* (Figure 4a; Figure S8). As MSCs commit to the adipocyte lineage, they undergo a series of orchestrated events leading to the development of mature adipocytes. Perilipin, a key protein associated with lipid droplets, plays a crucial role in adipocyte function and lipid metabolism. MSCs cultured in adipogenic conditions inside the different silk scaffolds differentiated into PERILIPIN^+^ adipocytes, demonstrating an accumulation of lipid droplets similar to that observed in standard cultures (Figure 4a; Figure S8). No differences were observed between S_SF10 and S_SF50 scaffolds, indicative of the maintenance of a comparable differentiation potential.

RNA sequencing (RNA‐seq) was performed to provide a quantitative snapshot of the transcriptome of MSCs from standard 2D cultures or the different 3D silk bone marrow models. Consistent with the morphological analysis and proven differentiation potential, RNA‐seq revealed no significant differences in the expression of stemness‐related genes (e.g., *CXCL12, NES, LEPR, THY1, CYP1B1, KITLG*) under all tested conditions. We used differential gene expression (DEG) profiling to identify genes with significant expression changes between S_SF10 and S_SF50, compared to the standard culture. The results of this analysis are visually represented using volcano plots (Figure 4b). The comprehensive overview of the transcriptomic changes identified a significantly larger set of differentially expressed genes in S_SF50, the softest scaffold. To gain deeper insights into the biological functions and processes influenced by these DEGs, we performed Gene Ontology (GO) analysis to categorize genes based on their associated biological processes, molecular functions, and cellular components. Several enriched GO terms were pivotal to collagen and proteoglycan deposition (Figure 4c). Particularly, in standard cultures in petri dishes, there was an upregulation of genes associated with ECM remodeling (e.g., *ADAMs, SPARC, LOXL4, LOX, CHSY3, HAPLN1*) and the synthesis of collagen‐containing ECMs (e.g., *COL1A1, COL4A1, COL4A2, COL8A2*), compared to S_SF50 but not compared to S_SF10 scaffolds (Figure 4d).

The secretion of ECM components by MSCs plays a crucial role in shaping their surrounding microenvironment. Changes in gene expression profiles driven by the mechanical properties of the culture substrate suggest that, on the stiffest substrates, MSCs may spontaneously deposit ECM enriched in collagenous components, resulting in the formation of aberrant microenvironments that fail to replicate the native bone marrow niche. 3D reconstruction of S_SF10 and S_SF50 scaffolds demonstrated that MSCs actively contribute to shaping their niche‐like microenvironment by secreting both TYPE I COLLAGEN and FIBRONECTIN (Figure 4e). Though the S_SF10 scaffolds promoted significantly increased deposition of TYPE I COLLAGEN, resulting in a shift of the collagen‐to‐fibronectin ratio (Figure 4f). MSC engraftment contributed minimally to the stiffness of both scaffolds, which remained in the order of magnitude of what we measured for the bare scaffolds (Figure 4g).

**FIGURE 5:**
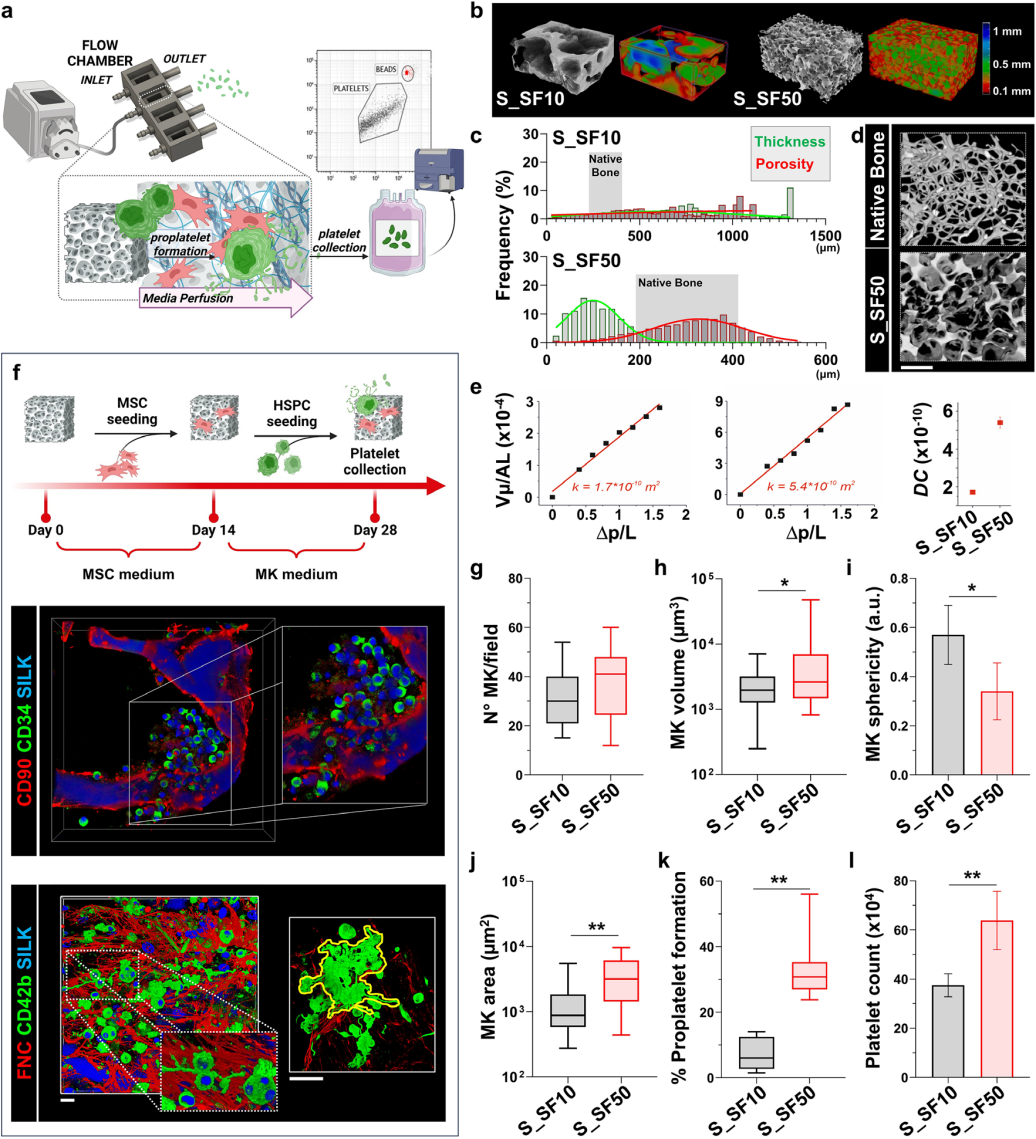
Characterization of silk scaffolds for *ex vivo* bone marrow models and HSPC differentiation toward megakaryopoiesis. (a) Schematic overview of the ex vivo perfusion chamber system having multiple chambers for culturing hematopoietic stem and progenitor cells (HSPCs) on silk fibroin (SF) scaffolds (S_SF10 and S_SF50). Media perfusion promotes platelet formation and collection in each independent chamber, allowing parallel analysis of different conditions. (b) Micro‐CT imaging of S_SF10 and S_SF50 scaffolds. Heatmaps represent porosity and thickness distribution. The 3D reconstructions emphasize structural differences between the scaffolds. (c) Frequency distribution of thickness (green) and porosity (red) for native S_SF10 or S_SF50 scaffolds. Grey squares highlight reference ranges for the native bone. (d) Micro‐CT images of native bone and salt‐leached scaffolds, illustrating comparable porosity and interconnectivity (scale bar = 500 µm). (e) Scaffold permeability analysis using Darcy's law. Linear fits of pressure drop against fluid velocity show increased permeability for S_SF50. Darcy coefficient values are quantified for both scaffolds (n = 3). (f) Experimental timeline for MSC and HSPC culture. MSCs were seeded on Day 0 for initial scaffold remodeling, followed by HSPC seeding on Day 14 and platelet collection on Day 28. Representative confocal reconstruction of the 3D co‐culture is shown. The upper panel shows CD34^+^ HSPCs (green) in close contact with CD90^+^ MSCs (red) (scale bar = 20 µm). The lower panel shows CD42b^+^ megakaryocytes (green) embedded within the fibronectin matrisome (red) after differentiation in the 3D co‐culture system (scale bar = 50 µm). (g) Quantitative analysis of megakaryocyte number per field of S_SF10 and S_SF50 scaffolds (data are presented as mean ± S.D. n = 50 per group. Statistical significance was determined using an unpaired two‐tailed Student's t‐test. p = NS). (h–j) Analysis of megakaryocyte volume, sphericity, and area on S_SF10 and S_SF50 scaffolds (data are presented as box‐and‐whisker plots (median, interquartile range, and min–max). n = 50 per group. Statistical significance was determined using an unpaired two‐tailed Student's t‐test. *p < 0.05, **p < 0.01). Data indicate scaffold‐dependent maturation effects. (k,l) Functional analysis of platelet formation. S_SF50 scaffolds demonstrate a significantly higher percentage of proplatelet‐forming cells (n = 7, **p < 0.01) and count of recovered platelets compared to S_SF10 (n = 4, **p < 0.01), reflecting S_SF50 scaffold suitability for hematopoietic differentiation (data are presented as box‐and‐whisker plots (median, interquartile range, and min–max) or mean ± S.D. Statistical significance was determined using an unpaired two‐tailed Student's t‐test. *p < 0.05, **p < 0.01).

Proper regulation of TYPE I COLLAGEN and FIBRONECTIN levels is critical for creating a microenvironment that supports platelet production [8, 23]. An unfavorable collagen‐to‐fibronectin ratio can hinder these processes, leading to suboptimal cellular responses. We therefore investigated whether the different 3D silk scaffolds could be conducive or not to megakaryopoiesis. To address this question, we developed a two‐stage workflow. Initially, we generated the MSC‐ECM microenvironment within the various silk scaffolds. This was followed by the addition of human adult HSPCs with concurrent induction of their differentiation into megakaryocytes in the presence of TPO. The resulting niches were then embedded in a modular flow chamber to induce platelet sprouting within a perfused flow medium, mimicking the bloodstream (Figure 5a). The permeability of the S_SF10 and S_SF50 scaffolds was assessed using micro‐CT imaging and Darcy coefficient calculations, demonstrating distinct structural and functional differences between the two scaffolds (Figure 5b–e). Micro‐CT reconstructions highlighted a denser structure of S_SF10 compared to the more open and porous architecture of S_SF50 (Figure 5b–d). Quantitative analyses of pore thickness and porosity distributions further confirmed these differences, with S_SF50 displaying larger and more interconnected pores (Figure 5c), comparable to those observed during the analysis of the native bone structure (Figure 5d). The same sieved NaCl porogen fraction (>500 µm) was used for all scaffold formulations, thus suggesting that structural differences of pore morphology and interconnectivity are not dictated solely by the porogen, but are also modulated by the intrinsic properties and assembly behavior of the different silk fibroin preparations. This further supports the ability to rationally tune silk bone marrow scaffolds through fibroin processing to meet distinct experimental requirements. The Darcy coefficient (k) revealed significantly greater fluid permeability in the S_SF50 scaffolds, a feature that supports enhanced nutrient exchange and promotes cell function within the thrombopoietic niche (Figure 5e). Immunophenotyping of the cells within scaffolds provided detailed insights into cellular behavior. CD34^+^ HSPCs were evenly distributed throughout the volume of the 3D scaffolds and formed close associations with CD90^+^ MSCs (Figure 5f). Over two weeks, HSPCs differentiated into polyploid megakaryocytes expressing lineage‐specific markers such as CD61 and CD42b, mimicking the phenotypic characteristics observed in native bone marrow (Figure 5f, lower panel, and Figure S9). The S_SF50 scaffolds showed a higher density of thrombopoietic niches, and a significantly increased megakaryocyte volume compared to S_SF10, along with decreased sphericity, indicative of advanced maturation and enhanced differentiation into platelet‐producing cells (Figure 5g–i). As shown in Figure 1j, the quantitative analysis of cell volume and area coverage provides critical insights into the stages of megakaryocyte maturation and their functional roles in thrombopoiesis. The S_SF50 scaffolds demonstrated enhanced proplatelet formation, characterized by increased cell volume, greater surface area coverage, and reduced sphericity, which reflected extensive branching of proplatelet shafts compared to S_SF10 (Figure 5j,k). Perfusion experiments revealed a significantly higher platelet yield from megakaryocytes cultured in the S_SF50 scaffolds (Figure 5l), highlighting their superior capacity to support physiologic thrombopoiesis and efficient platelet release. These findings highlighted that the S_SF50 scaffolds, with their open porous architecture and optimized collagen‐to‐fibronectin ratio, create a soft, biomimetic niche that supports megakaryopoiesis and promotes efficient platelet production.

### 2.5 Modeling Bone Marrow Fibrosis Using the Silk System

**FIGURE 6:**
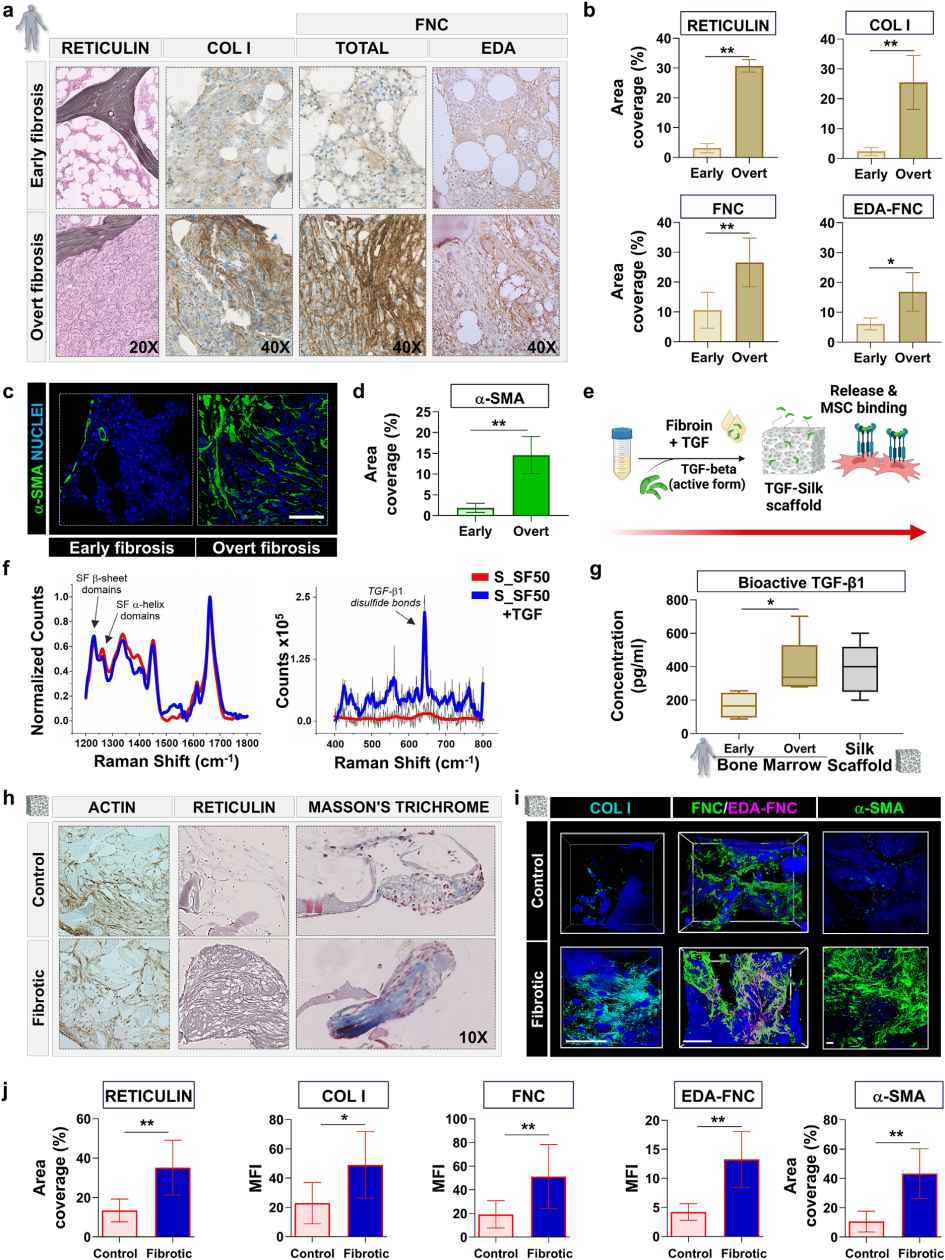
TGF‐β1‐functionalized silk scaffolds promote extracellular matrix remodeling and myofibroblast activation in a fibrotic bone marrow microenvironment. (a) Representative histological images of bone marrow biopsies from patients with early and overt fibrosis stained for reticulin, TYPE COLLAGEN I (COL I), total FIBRONECTIN (TOTAL FNC), and EDA‐containing FIBRONECTIN (EDA‐FNC), showing progressive extracellular matrix (ECM) accumulation (magnifications: 20X for reticulin, 40X for COL I and FNC). (b) Quantification of ECM components reveals significantly increased area coverage of reticulin, COL I, total FNC, and EDA‐FNC in overt compared to early fibrosis (data are presented as mean ± S.D. n = 25 per group. Statistical significance was determined using an unpaired two‐tailed Student's t‐test. *p < 0.05, **p < 0.01). (c) Immunofluorescence images of α‐SMA (green) and nuclei (blue) in early versus overt fibrosis bone marrow (scale bar = 50 µm) (d) Quantitative analysis of α‐SMA^+^ area confirms a significant increase in myofibroblast presence in overt fibrosis (data are presented as mean ± S.D. n = 25 per group. Statistical significance was determined using an unpaired two‐tailed Student's t‐test. **p < 0.01). (e) Schematic of the TGF‐β1 functionalization process: recombinant TGF‐β1 is loaded into silk fibroin (S_SF50) scaffolds, where it is stabilized and subsequently released to influence MSC behavior. (f) Raman spectroscopy of S_SF50 and S_SF50+TGF scaffolds highlights structural modifications upon TGF‐β1 incorporation, including peaks associated with β‐sheet domains and TGF‐β1 disulfide bonds. (g) Bioactive TGF‐β1 levels in bone marrow aspirates from early and overt fibrosis, and in S_SF50+TGF scaffolds, showing elevated levels in overt fibrosis and effective loading/release from scaffolds (data are presented as box‐and‐whisker plots (median, interquartile range, and min–max). n = 7 per group. Statistical significance between early and overt fibrosis was determined using an unpaired two‐tailed Student's t‐test. *p < 0.05). (h) Histological analysis of scaffolds (S_SF50, control vs. S_SF50+TGF, fibrotic) stained for actin, reticulin, and Masson's trichrome reveals increased fibrosis‐like ECM deposition in TGF‐β1‐loaded constructs (magnification: 10X). (i) Immunofluorescent staining of scaffolds for COL I, FNC/EDA‐FNC, and α‐SMA shows enhanced ECM accumulation and myofibroblast activation in fibrotic scaffolds (scale bar = 100 µm). (j) Quantification of reticulin, ECM markers, and α‐SMA expression demonstrates significantly higher matrix deposition and myofibroblast activation in fibrotic scaffolds (data are presented as mean ± S.D. n = 25 per group. Statistical significance was determined using an unpaired two‐tailed Student's t‐test. **p < 0.01).

To explore the pathological dynamics of Myeloproliferative Neoplasms (MPNs), a clonal stem cell disorder characterized by progressive bone marrow fibrosis, we employed our silk‐based bone marrow model to actively replicate and investigate the biochemical and cellular disruptions associated with human disease [45]. Patient‐derived samples from individuals with MPNs were used to establish parallels between early and advanced stages of primary myelofibrosis. Histological and immunohistochemical analyses of patient bone marrow specimens revealed significant changes in ECM composition, with the advanced fibrotic stage characterized by an increase in reticulin fibers, TYPE I COLLAGEN, FIBRONECTIN, alongside α‐SMA‐positive myofibroblast accumulation (Figure 6a;c). Quantitative analysis corroborated these findings, showing elevated levels of reticulin, TYPE I COLLAGEN, FIBRONECTIN, including the EXTRA DOMAIN A (EDA)‐FIBRONECTIN isoform typically expressed during fibrosis, and α‐SMA‐positive cells (Figure 6b;d). Transforming Growth Factor‐beta1 (TGF‐β1) plays a central role in the pathogenesis of bone marrow fibrosis in MPN progression. It is predominantly secreted by malignant megakaryocytes, driving the pathological remodeling of the bone marrow microenvironment. To recreate and probe these pathological features, we leveraged the incorporation of TGF‐β1 into S_SF50 scaffolds, referred to as S_SF50+TGF scaffolds (Figure 6e). Traditionally, TGF‐β1 is added in its active form to growth media for in vitro studies of fibrotic activation. However, this approach fails to fully represent the native cell‐ECM interactions that regulate growth factor release. Raman spectra within the ‘fingerprint’ spectral region (from 500 to 1800 cm^−1^ wavenumber shift) of the S_SF50+TGF scaffolds revealed characteristic peaks for β‐sheet domains at 1083, 1229, and 1663 cm^−1^ (Figure 6f). A relatively weak peak at 1265 cm^−1^ was associated with α‐helix domains. The integration of TGF‐β1 within the S_SF50+TGF scaffolds was analyzed by examining disulfide peaks. TGF‐β1 contains cysteine residues that form intramolecular disulfide bonds, known as cysteine knots, which are the typical structure of TGF‐β superfamily. Disulfide vibrational modes in the 500 to 550 cm^−1^ spectral range were detected only in S_SF50+TGF scaffolds, not in the S_SF50 scaffolds (Figure 6f). The peak around 503 cm^−1^ was attributed to the gauche‐gauche‐gauche configuration of disulfide bonds, while the smaller peak around 523 cm^−1^ was linked to the gauche‐gauche‐trans configuration. The lower presence of β‐sheets domains in SF50 may enhance the solvent accessibility of protein amine bonds, potentially improving the distribution homogeneity of the loaded drug during scaffold fabrication. Quantification of TGF‐β1 concentration in the culture medium showed a daily release, similar to the amount quantified in the bone marrow aspirate of our cohort of patients, during the overt fibrosis stages (Figure 6g). The controlled release of TGF‐β1 from the silk scaffolds was designed to provide a dynamic microenvironment, supporting MSCs and guiding them through fibrosis development before HSPC co‐culture. Indeed, human MSCs, integrated into S_SF50+TGF (fibrotic) scaffolds, demonstrated increased collagen deposition, as shown by reticulin and Masson's trichrome staining, compared to the S_SF50 (control) scaffold (Figure 6h). Additionally, there was a significant increase in the deposition of ECM components in the fibrotic scaffolds, including collagen type I, fibronectin, EDA‐fibronectin, and a marked increase in the number of α‐SMA^+^ cells (Figure 6i, Figures S10a and S10b). To define the response of the model to TGF‐β incorporation, we performed RNA‐seq analyses on MSCs cultured in the S_SF50+TGF scaffolds compared to those cultured in S_SF50. DEG and subsequent GO analyses revealed upregulation of genes for ECM component synthesis (e.g., *COL1A1, COL4A1, COL4A2, COL8A2, FBN1, VCAN, SDC1*), collagen synthesis and remodeling (e.g., *ADAMs, SPARC, LOXL4, LOX, CHSY3, HAPLN1*), and smooth muscle cell functions, indicative of myofibroblast trans‐differentiation (e.g., *ACTA2, ACTG2, COMP, COL1A1, HAPLN1, SERPINE1, TGFBI*) (Figure S10c–e). These changes corresponded to the stiffening of the scaffolds (Figure S10f). Despite these differences, the two niches supported comparable HSPC engraftment (Figure S11), indicating that the overall efficiency of the system, in terms of cell seeding and co‐culture capability, was equivalent between conditions.

### 2.6 A Divergence Index Uncovers How Fibrosis Shapes Megakaryocyte Dynamics

**FIGURE 7:**
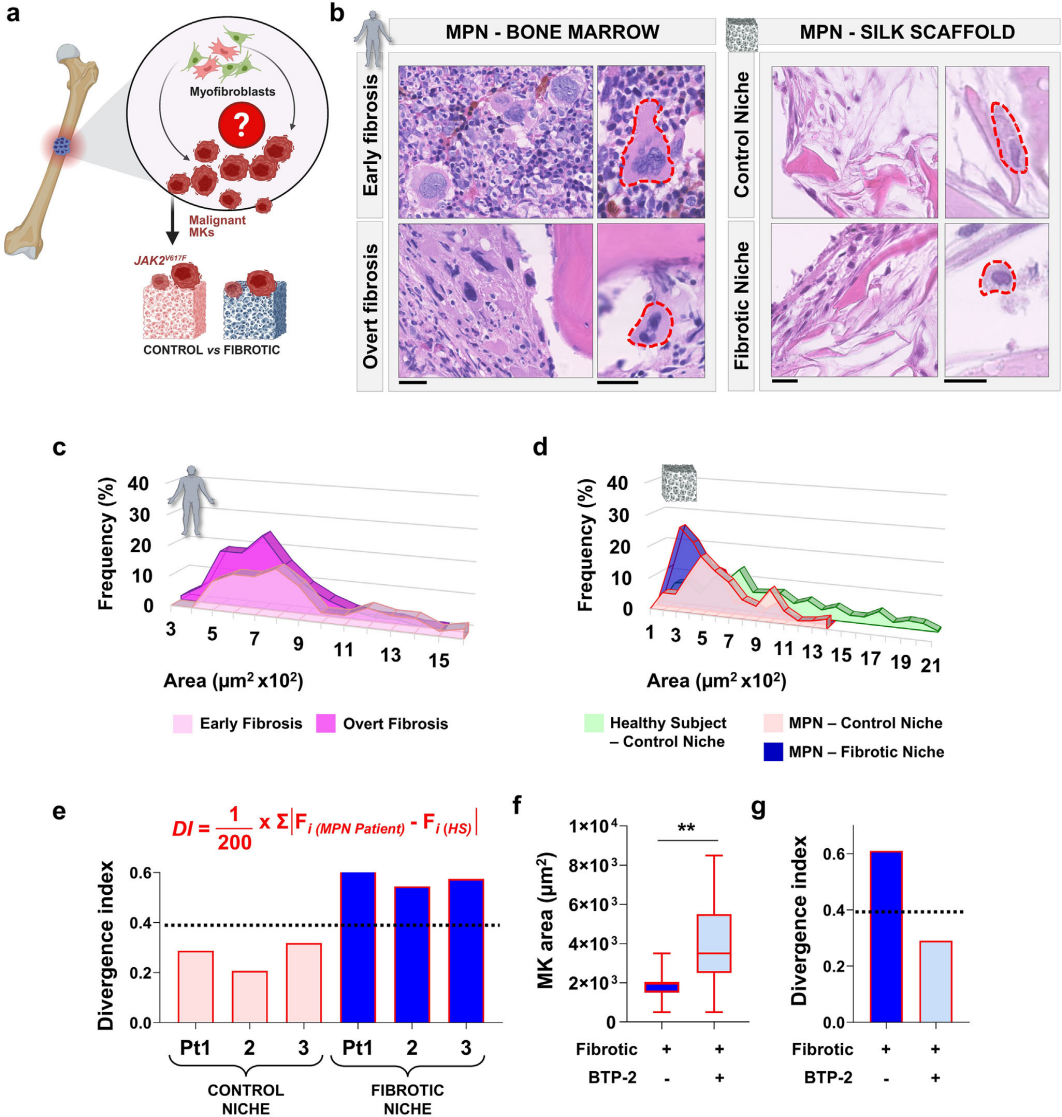
Reproducing aberrant thrombopoiesis in bone marrow fibrosis. (a) Schematic illustration of MPN‐associated bone marrow fibrosis. The specific impact of the niche on malignant megakaryocyte (MK) behavior, including proliferation and maturation, remains poorly understood. *JAK2^V617F^*‐mutated malignant megakaryocytes have been cultured within the two distinct scaffolds: control niche (non‐fibrotic) and fibrotic niche. (b) Bone Marrow: Histological analysis of bone marrow sections from patients with myeloproliferative neoplasms (MPN), showing megakaryocyte morphology in early versus overt fibrosis stages. Silk scaffold: Histological analysis of control versus fibrotic silk scaffolds cultured with MPN‐derived megakaryocytes (hematoxylin and eosin staining, scale bar = 25 µm). (c) Frequency distribution of megakaryocyte surface area in early (light pink) and overt (dark pink) fibrotic stages, showing a shift toward smaller megakaryocytes in overt fibrosis. (d) Frequency distribution of megakaryocyte area from n = 3 healthy subject samples (HS, green) cultured in the control niche, and n = 3 MPN samples cultured in control (red) versus fibrotic (blue) niches, confirming a fibrotic niche‐induced shift toward smaller megakaryocytes. (e) Implementation of a Divergence Index (DI) to quantify deviations in megakaryocyte size distribution between healthy and diseased states. DI values are elevated in MPN patients (Pt) cultured in the fibrotic niche, reflecting a higher divergence from healthy controls with respect to the same Pt, cultured in the control niche. (f) Quantification of megakaryocyte area in fibrotic conditions ± BTP‐2 treatment, showing partial restoration of megakaryocyte size with calcium modulation (data are presented as box‐and‐whisker plots (median, interquartile range, and min–max). n = 250 per group. Statistical significance was determined using an unpaired two‐tailed Student's t‐test. **p < 0.01). (g) Corresponding DI values from (f) indicate reduced divergence upon BTP‐2 treatment, consistent with improved megakaryocyte maturation.

To investigate the role of the microenvironment in myelofibrosis progression, we cultured *JAK2^V617F^*‐mutated megakaryocytes, derived from MPN patients, *ex vivo* within control silk scaffolds (S_SF50) or fibrotic scaffolds blended with TGF‐β1 (S_SF50+TGF) (Figure 7a). Histological comparisons between patient bone marrow biopsies and the silk‐based model revealed that the silk scaffold faithfully reproduces key pathological features of patient bone marrow, including reduction in megakaryocyte size within fibrotic niches (Figure 7b–d).

Within the silk scaffolds, frequency distribution analysis revealed a shift toward smaller megakaryocytes in MPN‐derived samples compared with healthy controls, particularly under fibrotic conditions, closely mirroring the size distributions observed in patient biopsies (Figure 7d). To quantitatively capture, *ex vivo*, the impact of niche composition on megakaryocyte size distribution, we introduced a Divergence Index (DI), calculated as the sum of absolute differences in class‐wise frequencies between conditions, normalized to a theoretical maximum (Figure 7e). A DI threshold of 0.4 defined the boundary between moderate and substantial divergence. DI values indicated moderate divergence in MPN samples cultured in control niches (DI ≈ 0.3), which increased markedly in fibrotic conditions (DI ≈ 0.6), highlighting the progressive impact of niche remodeling on megakaryocyte maturation. Store‐Operated Calcium Entry (SOCE) is the key regulator of megakaryocyte interaction with the bone marrow ECM environment, and its dysregulation is a hallmark of MPNs [46, 47]. To validate the model's responsiveness to calcium‐targeted therapies, we assessed the impact of SOCE inhibition on MPN‐derived megakaryocytes cultured within fibrotic scaffolds. MPN megakaryocytes exhibited pronounced calcium spikes and oscillations (Figure S12a), which were significantly dampened with BTP‐2, a selective SOCE inhibitor (Figure S12b). This pharmacological modulation led to a partial restoration of megakaryocyte maturation, evidenced by a significant increase in cell area (Figure 7f). This effect was quantitatively captured by a decrease in the DI from 0.61 to 0.29, corresponding to a 50% reduction in the deviation from the healthy distribution profile (Figure 7g). By integrating biomimetic scaffold design with quantitative readouts, we developed a versatile platform that replicates patient‐specific histopathology and captures how microenvironmental changes, such as fibrotic remodeling, affect megakaryocyte behavior. The addition of the Divergence Index provides a functional metric to assess disease progression and therapeutic response.

## 3 Conclusion

We developed an advanced bone marrow tissue model using silk fibroin‐based scaffolds that replicate the elastic properties and architecture of native bone marrow. By fine‐tuning silk cocoon degumming and controlling fibroin β‐sheet formation, we engineered a biomaterial that supports MSC function while preserving the biomechanical characteristics of the natural niche. MSCs cultured on soft silk scaffolds adhered effectively and remained undifferentiated over two weeks, maintaining their physiological characteristics. Only in these scaffolds, MSCs display gene expression profiles indicative of a healthy bone marrow microenvironment, with a well‐regulated ECM composition. When HSPCs were seeded onto the soft silk‐MSC scaffolds, they successfully differentiated into functional megakaryocytes in the presence of TPO. In contrast, differentiation was impaired in stiffer silk‐MSC co‐culture conditions. These findings highlight the critical role of scaffold compliance in preserving MSC function and facilitating hematopoiesis, positioning soft silk scaffolds as the ideal platform for physiological bone marrow modeling. To mimic bone marrow remodeling in myelofibrosis, we incorporated TGF‐β1 into the softest silk scaffold, driving MSC differentiation into myofibroblasts and promoting ECM deposition characteristic of a fibrotic matrisome. This bioengineered bone marrow niche captures the structural and molecular signatures of fibrosis observed in patient samples, offering a relevant environment to study hematopoietic dysfunction in a human‐like context. Despite carrying the same pathological mutations, patient‐derived HSPCs responded differently depending on the microenvironment, with the fibrotic niche inducing hyperproliferation, impaired maturation, and defective platelet production. These findings demonstrate that the microenvironment is not a passive background but an active determinant of disease phenotype. By decoupling genetic and extrinsic drivers, our approach reveals mechanisms of hematopoietic deregulation that are not observable in conventional models.

To quantitatively assess how far a given hematopoietic phenotype deviates from physiological conditions, we developed a Divergence Index, a multi‐parametric system that integrates cellular, molecular, and mechanical readouts across our engineered niches. This index enables the comparative evaluation of different bone marrow microenvironments and provides a quantitative scoring system to stratify disease severity and progression. By embedding this index into our model, we have created a scalable framework for mechanistic studies and preclinical testing, including patient‐specific disease modeling and drug screening. Built around HSPCs and MSCs, this platform enables modular reconstruction of marrow complexity, providing new opportunities for decoding the environmental cues that shape hematopoiesis in health and disease.

### 3.1 Experimental Section/Methods

#### 3.1.1 Materials. Main Chemicals, Biochemicals, and Other Materials are Listed in Table S2

### 3.2 Ethical Statements

Human samples from healthy subjects and MPN patients were collected at the foundation I.R.C.C.S. San Matteo, Pavia, and the foundation I.R.C.C.S. Ca’ Granda Ospedale Maggiore Policlinico, Milan. The study was approved by the Local Ethics Committees (20190024989, 20190105520). Written informed consent was obtained from all patients in accordance with the declaration of Helsinki. The diagnosis of MPN was established according to the 2016 revision of the World Health Organization Classification of Myeloid Neoplasms and Acute Leukemia [48]. Patients with a Fibrosis Grade of MF‐0/1 were classified as being in an early fibrotic stage, whereas those with MF‐2/3 were classified as having overt fibrosis. The patients were selected only if they carried the *JAK2* ^*V617F*^ mutation and were not receiving disease‐modifying therapies.

All animal studies were reviewed and approved by the Italian Ministry of Health (approval 990.2017‐PR/2017). C57BL/6J mice were from Charles River Laboratories. All animals were sacrificed according to the current European Legal Animal Practice Requirements. The tibia and femur of wild‐type mice were removed. Bone marrow samples were flushed out of the bones. Explants and bones were fixed overnight in 4% Paraformaldehyde (PFA) and then transferred to PBS for downstream analyses

### 3.3 Micro‐Computed Tomography (Micro‐CT)

For microCT (µCT) experiments, the bones were wrapped in gauze moistened with PBS, enclosed in parafilm, and fixed to a dedicated high‐resolution sample holder. µCT scans were carried out at a nominal pixel size of 4 µm by means of a Bruker SkyScan µCT 1276 scanner (Skyscan, Kontich, Belgium) at 55 kV and 72 µA, using a 0.25 Mm aluminum filter. Four images were captured and averaged every 0.2° through 180° of rotation at the maximum camera resolution of 4032 × 2688. The reconstruction was performed with the SkyScan NRecon 2.2 software using the InstaRecon reconstruction engine, ring artifact reduction, and beam hardening correction. Image analysis was carried out by means of the Skyscan CT Analyser 1.20.8.0+ (CTAn) software. A reference slice was established at the level of the growth plate of the proximal tibia and of the distal femur, respectively. Then, trabecular regions were extracted as follows: A volume of 1 Mm starting from 0.4 Mm in the direction of the metaphysis for the tibia, and a volume of 2 Mm slices starting from 0.36 Mm in the direction of the metaphysis for the femur. The trabecular volume of interest (VOI), enclosed by the endosteal surface, was selected by means of a semiautomatic approach based on thresholding. Trabeculae were binarized, keeping the same global threshold corresponding to a bone mineral density (BMD) range of 0.508–1.919 G.cm^−3^ calcium hydroxyapatite (CaHA), calibrated by reference phantoms. morphometric parameters of the trabecular VOI complying with the American Society for Bone and Mineral Research Guidelines [49], including porosity, were calculated in 3D using a double‐time cubes model. The trabecular thickness Tb.Th (mm) and separation Tb.Sp (mm) distributions were calculated using the local thickness method and corresponding images, color‐coded according to the Tb.Th and Tb.Sp distributions were produced.

Scaffolds were drained of excess liquid, wrapped in Parafilm, and fixed to the high‐resolution sample holder. Silk scaffolds were scanned with the same parameters used for the bone. Two images were captured and averaged every 0.1° through 360° with 2 × 2 camera binning. The reconstruction with NRecon was performed with the same approach used for the bone. Two parallelepipeds corresponding to subregions of 34,5 (S_SF10) and 62,7 (S_SF50) mm^3^, respectively, were extracted owing to the smaller pixel size. In the CTAn a global threshold was optimized for each scaffold to segment the material. The 3D analysis was performed following the same method used for the bone. Using the SkyScan CT‐Voxel (“CTVox”) Software, volume‐rendered 3D images were generated for trabecular regions and scaffolds, and an RGB transfer function was set to visualize Tb.Sp maps. A rendering of the 3D surface model of trabeculae was also performed with the Skyscan CT‐volume (“CTVol”) Software.

### 3.4 Silk Fibroin Degumming

*Bombyx mori* silk cocoons were from CREA (Council of Research in Agriculture and Analysis of Agricultural Economics, Padova, Italy). Samples were cut with stainless steel scissors and boiled for 10, 30, or 50 min in a glass cylinder containing 0.02 M Na_2_CO_3_ aqueous solution. The samples were respectively referred to as SF10, SF30, and SF50. The samples were then rinsed in 2.0 L of ultrapure water three times for 10 min while gently stirring and then left to dry at room temperature for three days. Then, SF was dispersed in LiBr aqueous solution, at 60°C for 4 h; the resulting solution was dialyzed (12 mL Slide‐A‐Lyzer dialysis cassette; MWCO 3,500 Da; Thermo Fisher Scientific, UK) against ultrapure water for three days, changing the ultrapure water four times per day, and finally centrifuging at 4,000 Rpm for 10 min to remove insoluble particulates. The final concentration was determined by freeze‐drying 0.5 mL of SF solution and thereby expressing the corresponding mass loss

### 3.5 Preparation of Silk Fibroin Scaffolds via Salt Leaching

A 20.0 × 8.0 × 4.0 Mm (L × W × H) rectangular parallelepiped‐shaped mold was filled with ∼600 µL of 8% Wt SF10 or SF50 aqueous solution and 2 Gr NaCl Particles (Sigma–Aldrich), sifted to have diameters greater than 500 µM [21, 39, 40]. Samples were left to dry at room temperature for two days; then the SF‐NaCl material was immersed in 0.5 mL ultrapure water to remove the salt. The water was replaced for two days, twice a day. The SF scaffolds prepared with SF10, SF30, and SF50 were labeled as S_SF10, S_SF30, and S_SF50. Scaffolds were sterilized with UV radiation.

### 3.6 Swelling Test. Scaffolds Were Placed in Deionized Water

The surface moisture was removed with a filter paper, and the weight of 5.0 × 5.0 × 4.0 Mm (L × W × H) hydrated samples (*W*_*h*_) was determined. Samples were then dehydrated using a Genevac EZ2 elite centrifugal evaporator (SP Scientifics, US) and re‐weighted (*W*_*d*_). The swelling, considered as the water absorption, was calculated according to the following equation:

(1) $$Swelling\;\%=\frac{W_{h}-W_{d}}{W_{d}}*100$$

### 3.7 Preparation of TGF‐β1‐Loaded Scaffolds and TGF‐β1 Release

TGF‐β1, previously reconstituted in citric acid, was physically blended into the SF50 aqueous solution to obtain a final S_SF50+TGF‐β1 formulation containing 8% wt. SF and 300 ng mL^−1^ TGF‐β1. The resulting solution was then used to cast a silk scaffold via salt leaching, following the method described above. The release of TGF‐β1 from the S_SF50 scaffold was monitored in culture medium after 30 min, 1 h, and each day up to 15 days of culture; different samples were used in triplicate for each time point, quantifying TGF‐β1 with the human TGF‐β1 ELISA Kit (Sigma–Aldrich, Germany), following the manufacturer's protocol

### 3.8 Raman Spectroscopy

Raman spectra of hydrated S_SF50 and S_SF50+TGF‐β1 scaffolds (5000 accumulation per sample) were acquired by means of invia renishaw raman microscope (Renishaw plc, UK), setting the laser excitation (excitation power 100 mW) at 785 Nm and the exposure time of 1s, and using a 1200 L Mm^−1^ Reticle.

### 3.9 Size Distribution in Water Dispersion. A)

Asymmetric flow field‐flow fractionation (AF4). 3 mg mL^−1^ SF10, SF30, or SF50 solutions in 1.0 mM LiBr were analyzed using an AF2000 TM AF4 System (Postnova Analytics, Landsberg, Germany) coupled to a PN3210 UV/Vis detector working at a wavelength of 220 Nm (Shimadzu SPD‐20A, Postnova Analytics), and a Dawn Heleos II Multi‐angle Light Scattering (MALS) (Wyatt Technology, Santa Barbara, California) working at a wavelength of 660 Nm and a Optilab T‐rEX refractive index (RI) (Wyatt Technology) detector in the given order. The AF4 Frit‐inlet channel was equipped with a 350 µm Spacer and a 10 kDa MWCO regenerated cellulose membrane as the accumulation wall. The detector flow rate was set at 0.5 mL min^−1^, and 50 µL of solution was injected at a flow rate of 0.2 mL min^−1^. In the subsequent elution, the cross flow was maintained constant at 2.0 mL min^−1^ for 0.2 min and then exponentially (exponent  =  0.20) decreased to 0.1 mL min^−1^ over 40 Min. The MALS and RI data were analyzed using the astra software (Version 7.3.2, Wyatt Technologies), fitting with a zimm model after subtracting a blank run obtained by injecting 50 µL of in 1.0 mM LiBr, and obtaining distributions for molar mass and radius of gyration (***R***_***g***_). B) Dynamic Light Scattering (DLS). DLS experiments on three independent samples were carried out on 1.0 mg mL^−1^ SF10, SF30, or SF50 in 1.0 mM LiBr, using a Möbiuζ instrument (Wyatt Technology) (λ = 532 nm, scattering angle of 163.5°), obtaining the Z‐average Radius (***R***_***h***_) and polydispersity (PDI).

### 3.10 Secondary Structure Determination

A) Attenuated total internal reflectance (ATR) infrared spectroscopy. 400–4000 cm^−1^ IR absorbance spectra (resolution 4 cm^−1^, 96 scans) were collected by depositing 1.0 mg mL^−1^ solutions of SF10, SF30, and SF50 in MilliQ water on the atr crystal of an alpha II FT‐IR spectrometer (Bruker, UK), using a background spectrum of deionized water. Since the Amide I band was heavily affected by the absorption of the water bending vibration, we have focused this analysis on the Amide II (1480–1580 cm^−1^) region. That band was fitted with two Gaussian curves, one (peaked at 1510–1520 cm^−1^) assumed proportional to the β‐sheet content of fibroin and the other (peaked at 1540–1550 cm^−1^) to the content of α‐helix secondary structures [50]. See Figure S1, for examples of such fittings. A crystallinity index (CI) was then calculated as the ratio between the two different forms of secondary structures

B) Fluorescence Spectroscopy. Thioflavin T (ThF‐T) was used as a dye for selectively staining the β‐sheet domains and relatively quantifying their content (please note that ThF‐T fluorescence intensifies upon binding β‐sheet domains [51, 52]). A 1.0 mM solution of ThF‐T was prepared by dissolving 1.6 mg of ThF‐T (5 µmol) in 5 mL of Milli‐Q water and filtering through a 0.22 mm PES filter (Thermo Fisher Scientific). The ThF‐T fluorescence assay was performed on both: 1) SF10, SF30, and SF50 aqueous solutions, and 2) S_SF10, S_SF30, and S_SF50 scaffolds.

For details:1) 100 mL of SF10, SF30, and SF50 aqueous solution (final concentration of 5.0, 2.5, 1.25, 0.675 mg mL^−1^) were placed in 96‐well flat bottom black plates (Thermo Fisher Scientific), supplemented with 100 mL of ThF‐T (final concentration of 1.0 or 5.0 µM), shaken for 10 s and kept at room temperature in the dark for two hours. ThF‐T fluorescence measurements were performed by means of BioTek Synergy H1 multi‐mode microplate reader (BioTek Instruments, Inc., Winooski, VT, USA), exciting the samples, previously equilibrated at 37°C for 15 min, at 440 nm and recording the emission spectra in the range of 470 to 700 nm. β‐sheet relative content was evaluated by comparing the sample fluorescence intensity at 480 nm. 2) Hydrated S_SF10, S_SF30, and S_SF50 scaffolds were immersed in a 5.0 µM ThF‐T aqueous solution at room temperature in the dark for two hours and then rinsed with Milli‐Q water three times for ten minutes each. Fluorescence measurements were performed by means of Nikon A1 Laser Scanning Confocal Microscope (Nikon, Japan) using a 40x water objective at a constant laser intensity and gain. The sample mean fluorescence intensity (n = 3) was measured by collecting the fluorescence intensity data of ten regions (50 µm^2^) and subtracting the background signal.

### 3.11 Mechanical Characterization

A) Diffusing Wave Spectroscopy (DWS). DWS analysis was carried out in transmission mode (multi‐tau duration 300 s, echo duration 30 s) using a DWS RheoLab (LS Instruments AG, Fribourg, Switzerland) with a 40‐mW diode laser operating at a wavelength of 685 Nm. Reference samples were prepared by introducing 0.9 mL of 0.22, 1.11, 2.22, or 4.44% wt. SF10, SF30, or SF50 in aqueous solution into 1.5 mL Eppendorf tubes, followed by the addition of 100 µL of a 10% wt. dispersion of 500 Nm in diameter polystyrene particles (LS Instruments AG, Fribourg, Switzerland) in Milli‐Q water; the solutions were homogenized via vortexing with a Reax top shaker (Heidolph Instruments, Schwabach, Germany) at 1,500 Rpm for 10 s and transferred to optical glass cuvettes with a thickness of 5 Mm. The temperature of the sample cell holder was maintained at 25°C, and a fixed time of 10 min was set to stabilize the sample temperature before the analysis. For any measurement, the mean free path *l* ^*^ and absorption length *l*_*a*_ were automatically determined. The mean square displacement (MSD) 〈*Δr* ^2^(*t*)〉 of tracer particles was measured using time correlation functions, extracting the storage modulus *G′*, loss modulus *G″* and complex modulus *G* ^*^.

B) Rotational rheology. S_SF10, S_SF30, and S_SF50 scaffolds were prepared via salt leaching in six‐well (∅ = 35 mm) plates as described below; after drying at room temperature, the whole plate was immersed in deionized water for 3 days to remove salts, exchanging it three times a day. After lifting the plate out and removing excess water, the samples were carefully handled with tweezers and transferred onto the lower plate of a Haake MARS III rotational rheometer (Thermo Scientific, Karlsruhe, Germany) operated at 37°C; a parallel upper plate with the same dimensions as the scaffold (∅ = 35 mm) was then lowered to a 1.0 mm gap. Storage (*G*′) and loss (*G*′′) moduli were measured at 1.0 Hz and a 0.02% strain, averaging the values over a 5 min period. 0.1 – 100 Hz frequency sweep tests we performed at a constant strain of 0.02%; 0.002 – 0.03% strain amplitude sweep tests were performed at a constant frequency of 1.0 Hz, defining the ultimate strain (limit of the linear viscoelastic region, LVR) as the strain with a 10% deviation *G*′ from its plateau value.

C) Atomic Force Microscopy (AFM) imaging and nanoindentation. JPK Nanowizard 3 AFM (JPK instruments, Germany) was used in combination of a CellHesion module and NIKON Ti inverted microscope (Nikon Instruments, Japan), employing 12PtIr400B RMN probes (tip shank length of 80 µm (± 25%), cantilever length of 400 µm (± 15%), cantilever width of 50 µm (± 15%), spring constant of 0.6 N/m (± 40%), frequency of 6.0 kHz (± 30%), tip radius of ∼ 20 nm) (Rocky Mountain Nanotechnology, LLC, United States). The cantilever was calibrated using the contact‐based method prior to each data collection. In detail, S_SF10, S_SF30, and S_SF50 (10.0 × 5.0 × 1.0 mm, LxWxH) were anchored on a Superfrost microscope slide 76 × 26 mm (Fisherbrand, Fisher Scientific) and covered with 10 mM PBS buffer at pH 7.4. Measurements were performed in the Quantitative Imaging (QI) Mode on 3–6 10 µm^2^ regions (64 × 64 pixels, n = 3), with setpoint = 15 nN, Z‐length = 5.0 µm, and pixel time = 500 ms.

For imaging, height channels were calculated from the extended curves, smoothing data with a 40‐pixel moving average. A plane fit operation was performed to remove background information from the height channel, which arises from an overall sample tilt. Line fitting was applied to correct the offset by fitting each scan line with a linear fit, which is then subtracted from the same scan line, independently of the adjacent scan lines. Images were finally subjected to a low‐pass filter by applying Gaussian convolution (degree of smoothing 800 to 1500 mpx). The root mean square (RMS) roughness was calculated from 2.0 µm^2^ surface area, considering a total area region of 10.0 µm^2^ (n = 3). Nanoindentation: Young's Moduli were extrapolated from force‐indentation curves using a Hertz model, and their surface distributions were then fitted with a Lorentzian function: $y=\frac{2A}{\pi }\;\frac{w}{4{\left(x-x_{c}\right)}^{2}+w^{2}}$, where *x* and *y* are the modulus values and their occurrence, *x_c_* is the number average Young's modulus, *w* the width of the distribution and *A* the area under the curve.

D) Compression test. The Young's Modulus of S_SF10, S_SF30, and S_SF50 and S_SF50+TGF‐β1 scaffold with or w/o cells was quantified by performing uniaxial static compression tests on an Instron dual column tabletop universal testing System 3365 (Instron, Norwood, MA, US) equipped with a 10 N load cell. A constant deformation rate of 1 mm min^−1^ was applied until 60% strain was reached. The Young's modulus was calculated in the linear deformation at 5–10% strain (n = 3).

### 3.12 Cell Culture

a) MSC expansion. Human MSCs were purchased from Lonza and cultured in DMEM, 10% MesenCult MSC Stimulatory Supplement, 1% L‐glutamine, 1% P/S, and 1% non‐essential amino acids, 50 µM ascorbic acid [53, 54]. MSCs (maximum passage 3, tested negative for mycoplasma) were cultured in silk scaffolds for 15 days before analysis or co‐culture experiments.

b) MSCs/megakaryocyte co‐culture. Silk fibroin scaffolds were first conditioned with MSCs for 15 days to allow stromal adhesion, extracellular matrix deposition, and stabilization of a supportive hematopoietic microenvironment. CD34^+^ hematopoietic stem and progenitor cells (HSPCs) were obtained using previously described methods [46, 55]. CD34^+^ cells were purified from human blood or bone marrow aspirates of healthy donor controls and patients by immunomagnetic separation. Human HSPCs were then seeded onto pre‐conditioned scaffolds (day 15) at the desired density, and cultured at 37°C in a humidified 5% CO_2_ atmosphere for additional 2 weeks in Stem Span medium supplemented with 10 ng mL^−1^ TPO and IL‐11, 1% L‐glutamine, and 1% penicillin‐streptomycin, to induce megakaryocyte differentiation.

c) Osteoblast differentiation. Osteoblasts were differentiated from human bone marrow MSCs using previously described methods [54]. Briefly, MSCs were seeded in six‐well culture plates or silk scaffold. After two weeks, MSCs were supplied with a chemical differentiation medium, consisting of DMEM High Glucose w Sodium Pyruvate and w/o L‐Glutamine, 10% FBS, 1% L‐glutamine, 1% PS, 1% non‐essential amino acids, 100 nM dexamethasone, 10 mM sodium β‐glycerol phosphate, and 50 µM ascorbic acid. The MSCs were left to differentiate into osteoblasts for 5 weeks, changing the medium twice a week.

d) Adipocyte Differentiation. Adipocytes were differentiated from human bone marrow MSCs. Briefly, MSCs were seeded in 6‐well culture plates or silk scaffolds. After two weeks, MSCs were cultured in adipogenic medium containing DMEM High Glucose w Sodium Pyruvate w/o L‐Glutamine, 1% L‐glutamine, 1% PS, 10% FBS, 0.5 mM isobutyl‐methylxanthine, 1 µM dexamethasone, 10 µM insulin, 100 µM indomethacin for three days and maintained in medium with 10% FBS and 10 µM insulin for one day. The treatment was repeated three times, after which the cells were maintained in DMEM with 10% FBS and 10 µM insulin until day 21.

### 3.13 Oil Red‐O Staining

After 21 days of adipogenic differentiation, the adipogenic medium was removed from the MSCs, and the cells were washed with 1 × PBS. The cells were then fixed in 10% formalin for 30 min. Following two additional washes with PBS, the cells were stained for 20 min with an Oil Red O solution, which was diluted 3:2 in water. After staining, the cells were briefly rinsed with 60% isopropanol for less than one minute and then washed twice with water. Red lipid droplets within the cells were visualized and photographed using an Olympus IX53 (Olympus Europa SE & Co. KG, Hamburg, Germany) bright‐field microscope.

### 3.14 Von Kossa Staining

After 5 weeks of osteogenic differentiation, the osteogenic medium was removed from the MSCs, and the cells were washed with 500 µL of PBS for 10 min. The cells were then fixed using 1 mL of 25% formaldehyde for 10 min. After fixation, they underwent a 2‐min wash with 1 mL of Milli‐Q water. Subsequently, the cells were incubated under UV light for 30 min with 1 mL of 2.5% silver nitrate. Following the incubation, the cells were washed three times for 10 min each with 1 mL of 5% sodium thiosulfate. Finally, the cells were photographed to document the results using an Olympus IX53 (Olympus Europa SE & Co. KG, Hamburg, Germany) bright‐field microscope.

### 3.15 Perfusion Bioreactor Design

The bioreactor was designed using Fusion 360 (Autodesk Inc., San Rafael, California, USA) software and manufactured using the Form 3B+ 3D printer and BioMed Clear Resin (Formlabs, Somerville, Massachusetts, USA). The system consists of a rectangular block of 15.0 × 50.0 × 11.0 mm (LxWxH) with four wells of 7.0 × 5.0 × 6.0 mm. Perfusion tubes were positioned on either side of the bioreactor chambers and were connected to a perfusion system. The perfusion system was composed of a peristaltic pump (Shenchen Precision Pump, Baoding Peristaltic Pump Co., Ltd, Baoding, China) set at 90 µL min^−1^ and connected to the bioreactor inlet and a blood collection bag containing acid‐citrate‐dextrose (ACD) connected to the bioreactor outlet. The niches were perfused for 4 h [39, 40].

### 3.16 Permeability Analysis

Silk scaffolds were cast into custom cartridges measuring approximately one hundred eighteen millimeters in length, thirty point five millimeters in width, and thirty‐seven point five millimeters in height (Cellink, Sweden). Each cartridge was filled with two milliliters of a sixty‐six percent weight aqueous glycerol solution and connected to the pneumatic‐driven extrusion system of a Bio X 3D printer (Cellink, Sweden). The time required to perfuse the two milliliters of glycerol through the scaffold was recorded under varying applied pressures, including three, five, six, seven, and eight kilopascals.

Scaffold permeability was calculated using Darcy's law, where the flow rate (Q) is defined as the volume of fluid perfused over time. The equation considers the scaffold permeability (k), the cross‐sectional area (A), the applied pressure difference (Δp), the fluid viscosity (µ), and the scaffold height (L). The viscosity value used corresponds to that of a 66% wt. glycerol aqueous solution.

### 3.17 Imaging

Femurs were removed from six to eight‐week‐old mice, fixed for 24 h in PFA 4% and decalcified in a solution of EDTA 10%, in phosphate‐buffered saline (w/o calcium and magnesium) pH 7.2, for 2 weeks at 4°C. Specimens were then embedded in Optical coherence tomography (OCT) cryosectioning medium and snap frozen in a chilling bath. Eight µm‐tissue sections were prepared by using a Microm Microtome HM 250 (Bio Optica S.p.A, Milan, Italy, www.bio‐optica.it). For immunofluorescence staining, sections were fixed for 20 min in 4% PFA and blocked with 2% bovine serum albumin (BSA) in PBS for 30 min. Nonspecific binding sites were saturated with a solution of 5% goat serum, 2% BSA, and 0.1% glycine in PBS for 1 h. Specimens were incubated with primary antibodies anti‐fibronectin, and anti‐type I collagen, and incubated overnight at 4°C in washing buffer (0.2% BSA, 0.1% Tween 20 in PBS). After three washes, sections were incubated with appropriate fluorescently‐conjugated secondary antibodies in washing buffer for 1 h at room temperature (RT). Nuclei were counterstained using Hoechst 33258 (100 ng/mL in PBS) at RT for 3 min. Sections were then mounted with micro‐cover glass slips using Fluoro‐mount (Bio‐optica, Milan, Italy, www.bio‐optica.it).

Alternatively, mouse bone marrow explants were fixed for 20 min in 4% PFA, immediately after flushing. Samples were then washed with PBS. Specimens were incubated with FITC‐conjugated anti‐Cd41 antibody and primary antibody anti‐fibronectin, and incubated overnight at 4°C in washing buffer (0.2% BSA, 0.1% Tween 20 in PBS). After three washes, samples were incubated with appropriate fluorescently conjugated secondary antibodies in washing buffer for 1 h at room temperature (RT). Nuclei were counterstained using Hoechst 33258 (100 ng mL^−1^ in PBS) at RT for 3 min. Samples were then positioned onto glass coverslips and immediately imaged.

SF scaffolds were fixed and stained as previously described [39, 40]. Samples were probed with the primary antibodies of interest. Then, all samples were immersed in Alexa Fluor secondary antibody. Nuclei were stained with Hoechst 33258. Negative controls were routinely performed by omitting the primary antibodies. Images were acquired using the Olympus BX51 fluorescence microscope (Olympus Deutschland) and X10 or X20 Olympus UplanF1 objectives or with a TCS SP8 confocal laser scanning microscope (Leica, Heidelberg, Germany) equipped with X25 water‐immersion objective and X20 or X40 oil‐immersion objectives. 3D reconstruction, image processing, thresholding, and analysis were performed using Leica LasX (Leica), ImageJ/Fiji (NIH), and/or Arivis Vision 4D (Zeiss). Sphericity, area, and volumes were measured by Arivis Vision 4D (Zeiss) or ImageJ/Fiji (NIH).

Scanning electron microscopy (SEM) was performed as previously described [56].

Live calcium imaging and modulation of calcium flows have been performed as previously described [46, 47].

### 3.18 Flow Cytometry

MSC characterization was determined by staining the cells after 15 days of culture with CD90‐APC‐A750 and CD105‐PC7. Ex vivo produced platelets were gated using the forward/side scatter profile of donor platelets and identified as CD61^+^CD41^+^CD42b^+^ events, as previously described [39]. Isotype controls defined background. TruCount beads were used to quantify numbers. All samples were acquired with a BD FACSLyric Flow Cytometry System. Off‐line data analysis was performed using the Beckman Coulter Kaluza software package.

### 3.19 PCR and Real‐Time PCR

Retro transcription (RT) was performed in a final volume of 20 µL reaction using the iScriptTM cDNA Synthesis Kit according to the manufacturer's instructions. For quantitative real‐time PCR, RT samples were diluted up to 60 µL with bidistilled water, and 3.0 µL of the resulting cDNA was amplified in duplicate in 15 µL reaction mixture with 200 nM of each specific primer and SsoFast Evagreen Supermix at 1x as final concentration. The amplification reaction was performed in a CFX Real‐time system (BioRad Laboratories Inc. Hercules, CA, USA) as follows: 95°C for 5 min, followed by 35cycles at 95°C for 10 s, 60°C for 15 s, and 72°C for 20 s. Pre‐designated KiCqStart primers for *ALPL*, *SPP1*, *RUNX2*, and *Β2‐MICROGLOBULIN* genes were purchased from Sigma–Aldrich (Milan, Italy) (Table 3). *B2M* gene expression was used as housekeeping.

**TABLE 3:** List of primers.

Gene	Forward primer	Reverse primer
*RUNX2*	*AAGCTTGATGACTCTAAACC*	*TCTGTAATCTGACTCTGTCC*
*SPP1*	*GACCAAGGAAAACTCACTAC*	*CTGTTTAACTGGTATGGCAC*
*ALPL*	*ATCTTTGGTCTGGCCCCCATG*	*ATGCAGGCTGCATACGCCAT*
*B2M*	*AAGGACTGGTCTTTCTATCTC*	*GATCCCACTTAACTATCTTGG*

### 3.20 Bulk RNA Sequencing (RNA‐seq)

Nucleic acid extraction and purification were performed by RNA Extraction Kit (Qiagen). RNA samples were quantified, and their integrity (RIN, RNA integrity number) was assessed using High Sensitivity RNA ScreenTape Assay on 4200 TapeStation System (Agilent Technologies). For this method, 500 picogram RNA was used from each sample for cDNA generation using the Takara Smart‐Seq v4 Ultra Low Input RNA kit (Takara Bio USA, Ann Arbor, MI) following the manufacturer's instructions, with 15 cycles of cDNA amplification. This technology relies on the template‐switching activity of the reverse transcriptase to enrich for full‐length cDNAs and to add defined PCR adapters directly to both ends of the first‐strand cDNA. This ensures that the final cDNA libraries incorporate the 5′ end of the mRNA and maintain a true representation of the original mRNA transcripts. Smart‐Seq cDNA was assessed for quality on High Sensitivity D5000 Screen Tape Assay on 4200 TapeStation System. Starting from 500 cDNA picogram, libraries were prepared through DNA library prep Illumina kit (Illumina, San Diego, CA, USA), following manufacturer's instructions. This protocol is based by tagmentation drive on transposase cutting the genomic DNA and attaching the adapters on each side.

Final libraries were quantified (High Sensitivity D5000 Screen Tape Assay) and pooled at 0.8 nM concentration, then sequenced 1 × 100 bp on the Illumina NovaSeq 6000 platform.

Single‐end reads (100 bp) were trimmed with Trimmomatic (v0.39) [57]. to remove adapters and exclude low‐quality reads from the analysis and aligned to the GRCh38 reference genome using STAR aligner (v2.5.3a) [58]. (Dobin et al., 2013). FeatureCounts (v1.6.4) [59]. was used to compute reads mapping on exons as annotated in GENCODE Human basic gene annotation (version 31) and obtain transcript quantification summarized at the gene level, excluding multimapping reads.

Only genes showing an average greater than 1 CPM (Counts per million) were retained. Differential gene expression analysis was performed with DESeq2 Bioconductor library (v1.30.1) [60]. Hierarchically clustered heatmaps were made with Complex Heatmap R package (v2.11.1) [61]. GO analysis was conducted with Enrichr on DEGs (|log2FC|>0.5, adj.p‐value < 0.05) [62].

### 3.21 Immunohistochemistry

Bone marrow biopsies (BMBs) from Primary Myelofibrosis patients were obtained from the posterior superior iliac spine and decalcified using an EDTA‐HCL solution for 6 h. BMBs were formalin‐fixed and decalcified. Silk scaffolds were formalin‐fixed. All samples were paraffin‐embedded. Immunohistochemistry was performed using an automated staining system (BenchMark ULTRA; Ventana; Roche), as previously described [63]. Digital color RGB (red, green, blue) images per stained bone marrow sections from patients at different fibrotic phases of the disease were captured, converted to grayscale, subjected to thresholding, and analyzed with ImageJ to obtain the final staining measurement (expressed as percentage of total area).

### 3.22 Statistics

Values are expressed as mean plus or minus standard deviation or median and range. The Student t‐test was used to analyze experiments. A p‐value of at least 0.05 was considered statistically significant. All experiments were independently repeated at least three times, unless otherwise specified. GraphPad Prism or OriginPro softwares have been used for graph representation and statistical analysis.

## Funding

This paper was supported by the EIC Transition Project SilkPlatelet (Project n. 101058349), the European Hematology Association Advanced Research Grant (RG‐202012‐00212), Associazione Italiana per la Ricerca sul Cancro (AIRC) (Investigator Grant #18700), Italian Ministry of University and Research (PRIN 2022‐2022P9RM9M).

## Conflicts of Interest

The authors declare no conflicts of interest.

## Supporting information




**Supporting File**: smll72620‐sup‐0001‐SuppMat.pdf.

## Data Availability

Data supporting the results of this study are available within the paper. All raw and analyzed datasets generated are available from the corresponding author upon reasonable request.
