# Supplementary material for: Thermo‐Chemically Modified Silk Scaffolds Reveal Niche‐Driven Regulation of Hematopoiesis and Fibrosis
Source: Small. 2026 Jan 30;22(18):e13071. doi: 10.1002/smll.202513071 (PMC13014222; doi:10.1002/smll.202513071)
Supplement: Supplementary file 1 — Supporting File: smll72620‐sup‐0001‐SuppMat.pdf. [file SMLL-22-e13071-s001.pdf]

Supporting Information

THERMO-CHEMICALLY MODIFIED SILK SCAFFOLDS REVEAL NICHE-DRIVEN REGULATION OF HEMATOPOIESIS AND FIBROSIS

Christian A. Di Buduo, Carolina P. Miguel, Giulia Della Rosa,* Vittorio Abbonante, Santo Diprima, Delfina Tosi, Marta Filibian, Daniele Cattaneo, Jugal Kishore Sahoo, Nicola Tirelli, Alessandra Iurlo, Umberto Gianelli, David L. Kaplan, Alessandra Balduini* [¥]

C.A. Di Buduo, C.P. Miguel, G. Della Rosa, V. Abbonante, **A. Balduini**
Department of Molecular Medicine, University of Pavia, 27100, Pavia, Italy.
E-mail: alessandra.balduini@unipv.it

G. Della Rosa, N. Tirelli
Polymers and Biomaterials Lab, Istituto Italiano di Tecnologia, 16152, Genova, Italy.

S. Diprima
Center for Omics Sciences, IRCCS San Raffaele Scientific Institute, 20132, Milan, Italy.

D. Tosi, U. Gianelli
[4] Department of Health Sciences, University of Milan, Milan, Italy; Unit of Pathology, ASST Santi Paolo e Carlo, 20146, Milan, Italy.

M. Filibian
Centro Grandi Strumenti, University of Pavia, 27100, Pavia, Italy.

D. Cattaneo, A. Iurlo
Hematology Division, Foundation IRCCS Ca' Granda Ospedale Maggiore Policlinico, 20122, Milan, Italy.

D. Cattaneo, A. Iurlo
Department of Oncology and Hemato-Oncology, University of Milan, 20122, Milan, Italy.

J.K. Sahoo, D.L. Kaplan, **A. Balduini**
Department of Biomedical Engineering, Tufts University, 02155, Medford, MA, USA.
E-mail: alessandra.balduini@tufts.edu

¥ **Contact information for correspondence:** Alessandra Balduini, Department of Molecular Medicine, University of Pavia, Viale Golgi n. 19, 27100, Pavia, Italy. e-mail: alessandra.balduini@unipv.it and Department of Biomedical Engineering, Tufts University, Medford, MA, USA. e-mail: alessandra.balduini@tufts.edu

* These authors equally contributed.

Fundings: This paper was supported by the EIC Transition Project SilkPlatelet (Project n. 101058349), the European Hematology Association Advanced Research Grant (RG-202012-00212), Associazione Italiana per la Ricerca sul Cancro (AIRC) (Investigator Grant #18700), Italian Ministry of University and Research (PRIN 2022-2022P9RM9M).

Keywords: bone marrow, fibrosis, megakaryocytes, mesenchymal stem cells, platelets, silk fibroin, transforming growth factor beta1

Supplemental Figures and Tables

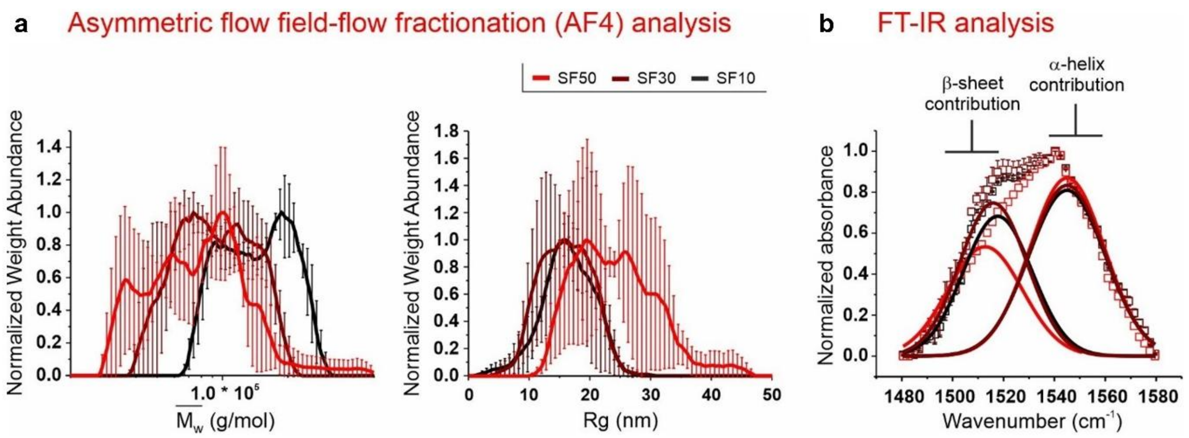

Figure S1. **(a)** Weight-average molar mass ($\overline{M_w}$, *left*) and radius of gyration (R_g, *right*) distribution of silk fibroin degummed for 10, 30 and 50 minutes (named as SF10, SF30 and SF50) obtained *via* asymmetric flow field flow fractionation (n=3). **(b)** FT-IR spectra in the region of amide II band (1480^{-1} - 1580 cm^{-1}) of SF10, SF30 and SF50 in aqueous solution.

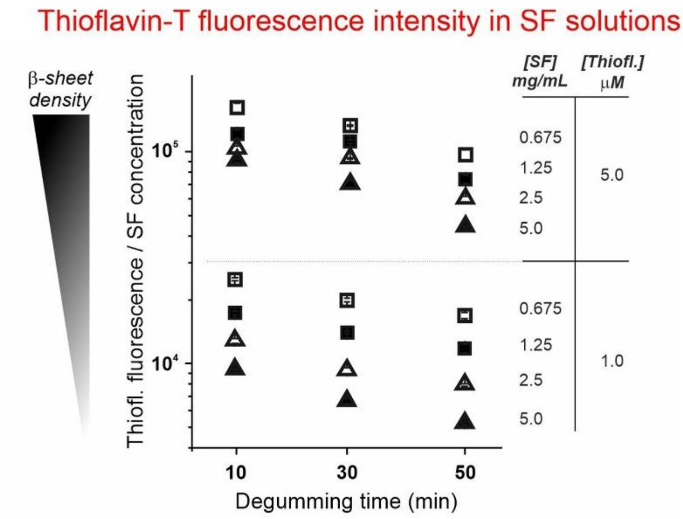

Figure S2. Thioflavin-T (1.0 and 5.0 μM) fluorescence intensity as a function of SF10, SF30 and SF50 concentration (0.675, 1.25, 2.5, 5.0 mg/mL).

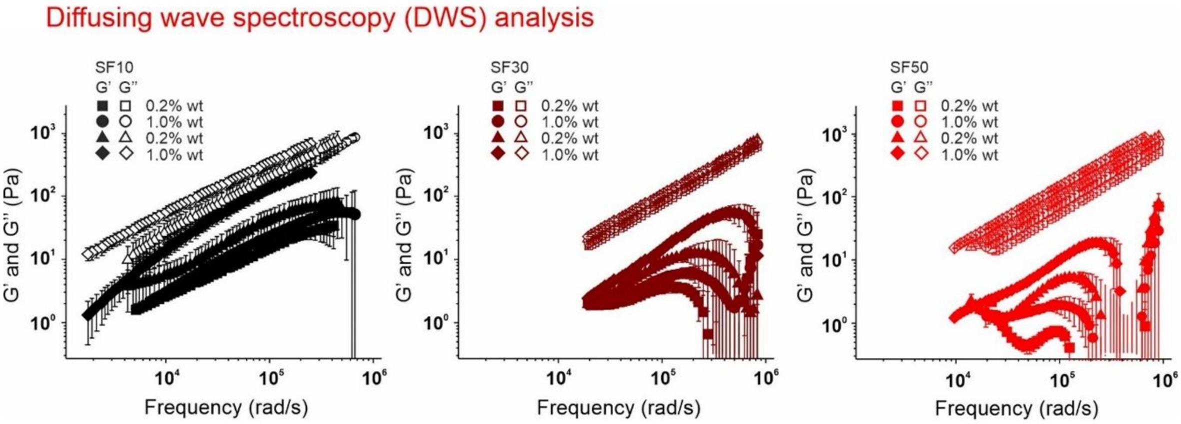

Figure S3. Storage moduli (G') and loss moduli (G'') from diffusing wave spectroscopy (DWS) analysis for 0.2 – 1.0 – 2.0 – 4.0 % wt SF10 (black symbols), SF30 (wine symbols) and SF50 (red symbols) as a function of frequency.

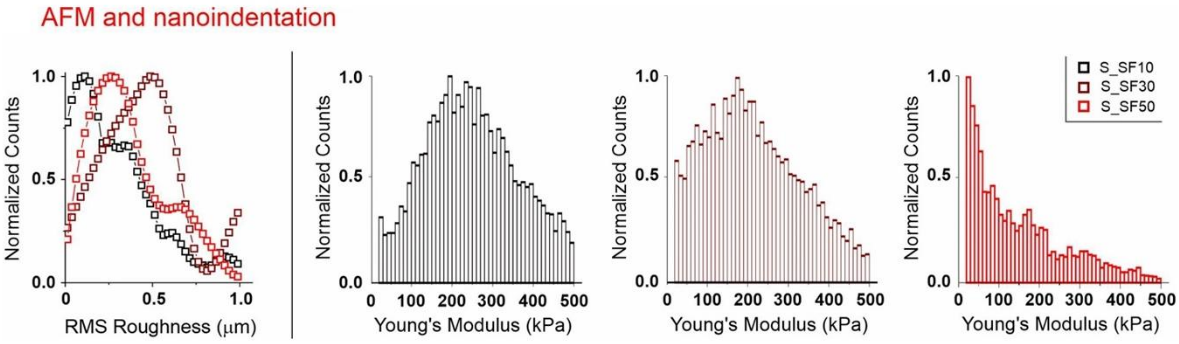

Figure S4. Distribution of and Root Mean Square (RMS) roughness (*left*) and Young's modulus (*right*) calculated from the force maps by means of AFM nanoindentation for S_SF10, S_SF30 and S_SF50 scaffolds. Young's modulus and RMS data were obtained by measuring 3-6 different, 10*10 μm areas (providing 100 Young's modulus data - each of them corresponding to a 1 μm^2 area - and 25 RMS data - each of them corresponding to a 4 μm^2 area).

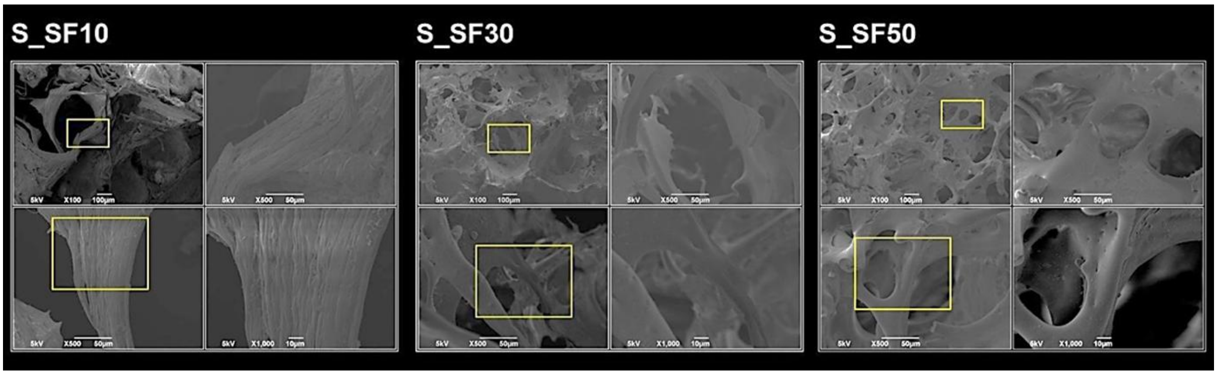

Figure S5. Higher (*top*) and lower (*bottom*) representative scanning electron microscopy (SEM) pictures for the three SF scaffolds.

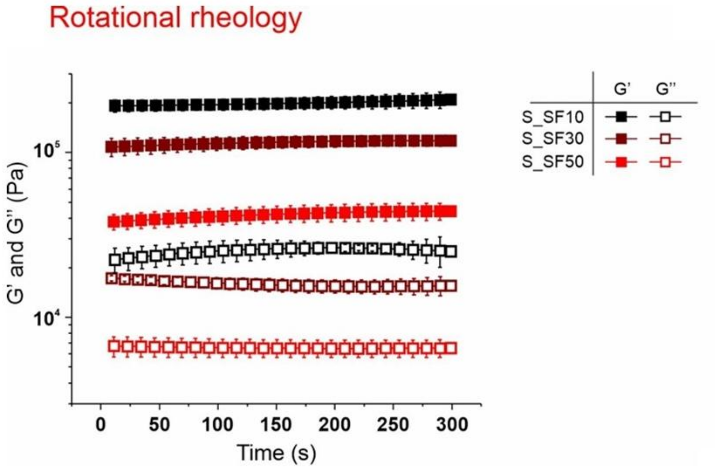

Figure S6. Storage (G') and loss moduli (G'') obtained *via* rotational rheology (0.02 strain, 1.0 Hz frequency) on scaffolds, which were transferred to the rheometer at the end of the washing phase; the upper plate was lowered until a normal force of 0.01-0.05 N was reached, and the measurements were started 5 minutes after.

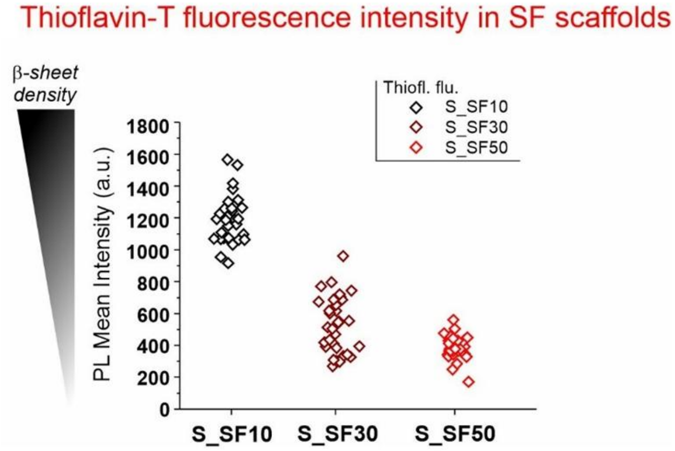

Figure S7. Mean fluorescence intensity of S_SF10, S_SF30 and S_SF50 stained with 5.0 μM thioflavin-T (ThF-T).

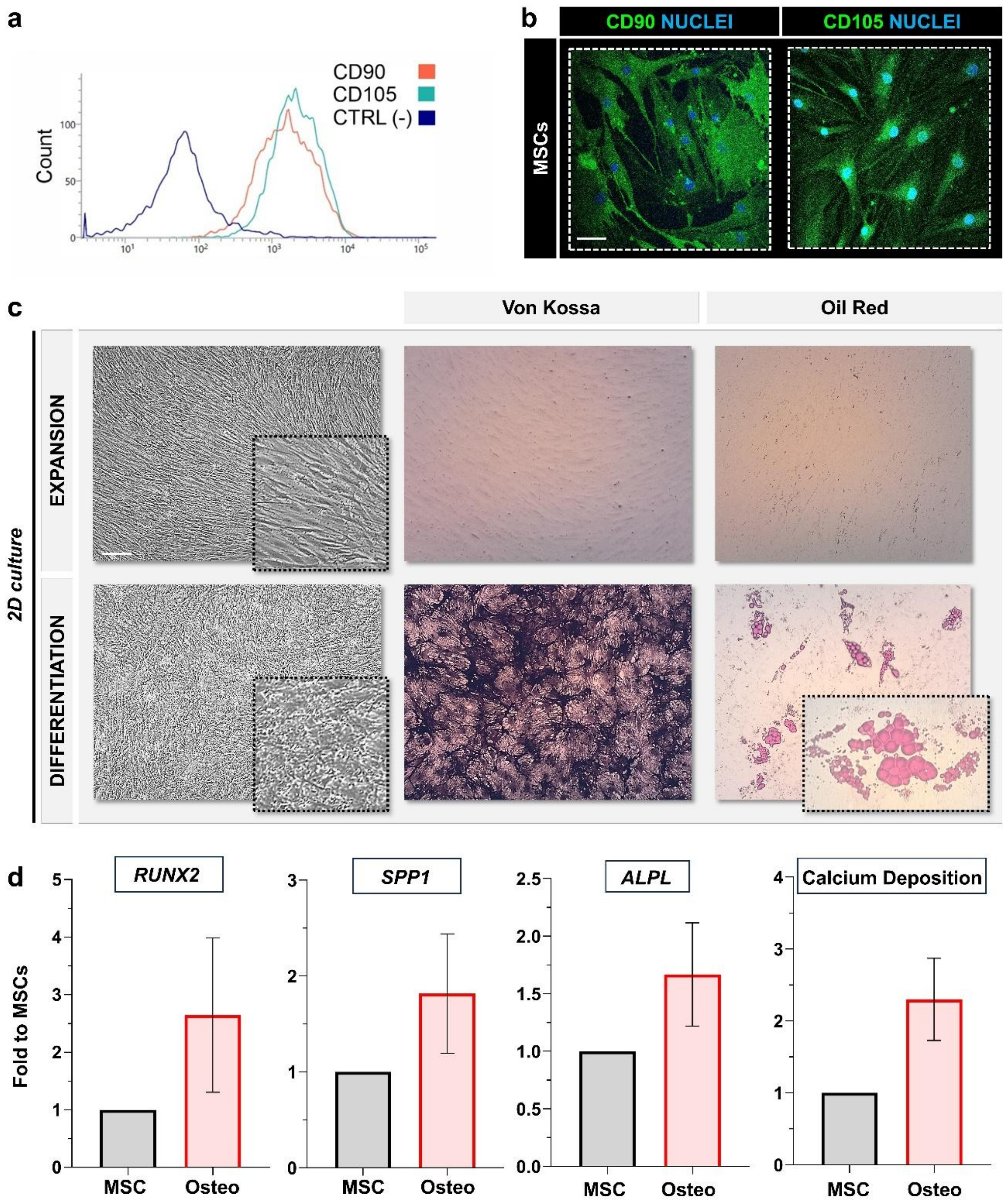

Figure S8. (a) Flow cytometry analysis of MSCs, demonstrating the expression of CD90 (orange peak) and CD105 (cyan peak) compared to a negative control (blue peak). **(b)** Immunofluorescence staining confirmed the expression of CD90 and CD105 (green) with nuclei counterstained (blue) in MSCs (scale bar = 30 μm). **(c)** MSC differentiation into

osteoblasts and adipocytes. The Von Kossa staining shows mineralized matrix deposition, indicative of osteogenic differentiation, while Oil Red O staining reveals lipid droplet formation, characteristic of adipogenic differentiation. Panels labeled "*Expansion*" show undifferentiated MSCs, and panels labeled "*Differentiation*" show the respective differentiated states (scale bar = 150 μm). **(d)** Gene expression analysis of osteogenic markers (*RUNX2*, *Spp1*, and *ALPL*), and calcium deposition levels in differentiated osteoblasts (Osteo) compared to undifferentiated MSCs (MSC). Data are presented as fold changes relative to MSCs, indicating increased expression of osteogenic markers and enhanced calcium deposition in osteoblasts (data are presented as mean±S.D. n = 3).

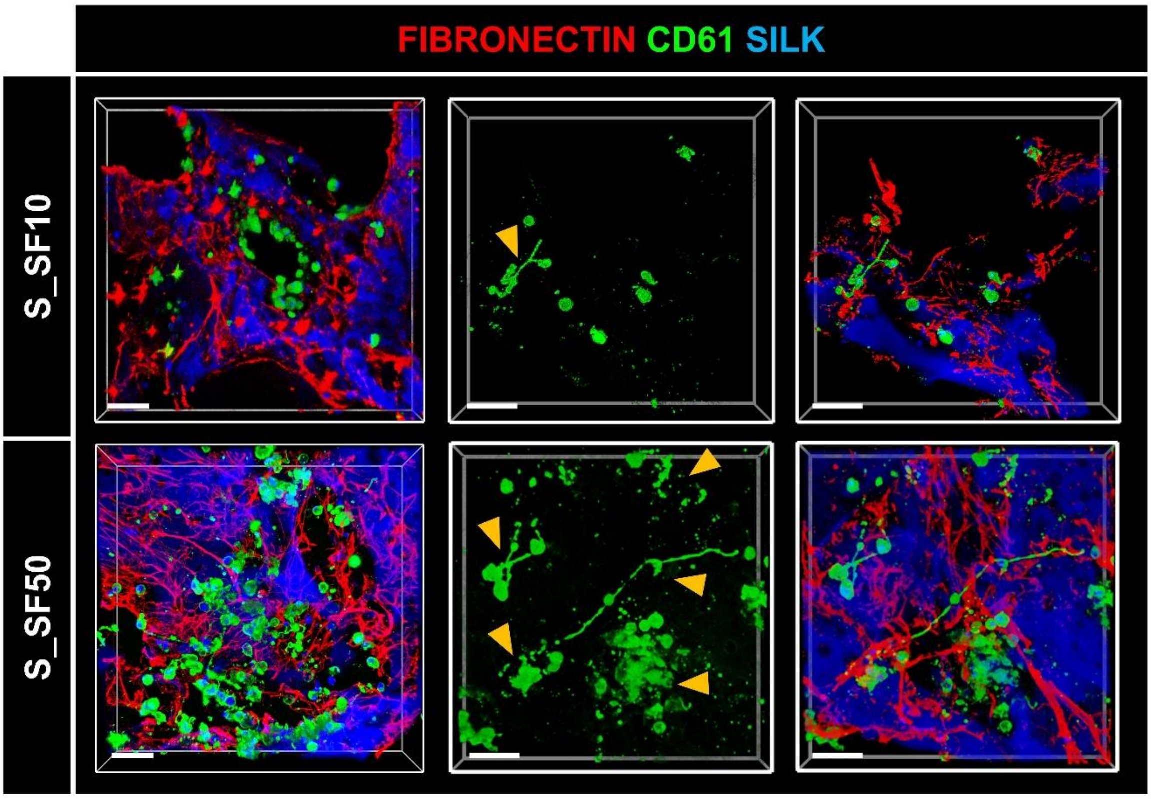

Figure S9. Fluorescence microscopy images display the organization and interaction of fibronectin (red), CD61-positive cells (green), within the different silk fibroin scaffolds (blue). The panels show various perspectives of 3D cultures. Yellow arrows indicate proplatelet-forming megakaryocytes (scale bars = 50 µm). Increased proplatelet formation and branching are visualized in S_SF50 scaffolds.

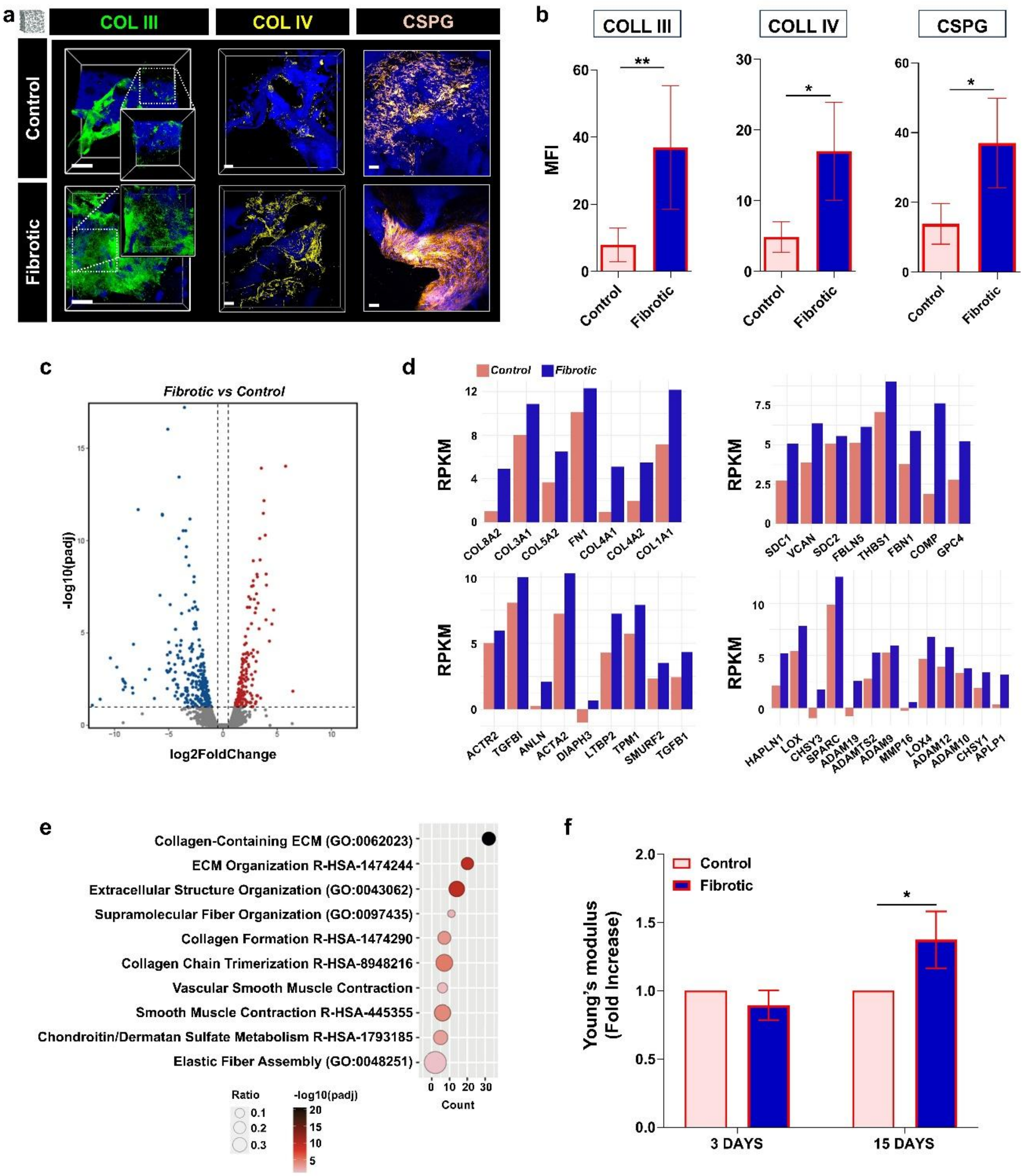

Figure S10. (a) Immunofluorescence images showing deposition of COLLAGEN TYPE III (COL III, green), COLLAGEN TYPE IV (COL IV, yellow), and CHONDROITIN SULFATE PROTEOGLYCANS (CSPG, orange) in Control (S_SF50) and Fibrotic (S_SF50+TGF-β1) scaffolds. Enhanced deposition of COL III and COL IV, and increased CSPG signal are observed in the fibrotic condition (scale bars = 50 μm). **(b)** Quantification of mean fluorescence intensity (MFI) for COL III, COL IV, and CSPG. TGF-β1 functionalization significantly

increases ECM component deposition in the fibrotic scaffolds compared to the bare control scaffolds (data are presented as mean±S.D. n = 25 per group. Statistical significance was determined using an unpaired two-tailed Student's t-test. $*p<0.05$, $**p<0.01$). **(c)** Volcano plot showing differentially expressed genes between Fibrotic and Control scaffolds. Upregulated genes are in red, downregulated genes are in blue. **(d)** Reads Per Kilobase of transcript per Million mapped reads (RPKM) of selected genes for ECM components, matrix regulators, proteoglycans, and signaling molecules, comparing Control (red) and Fibrotic (blue) scaffolds. **(e)** GO and pathway enrichment analysis of upregulated genes in the Fibrotic *vs* Control scaffolds. Terms related to collagen organization, ECM structure, and myofibroblast differentiation are significantly enriched. Circle size reflects gene count; color indicates adjusted p-value. **(f)** Young's modulus of the scaffolds, measured at 3 and 15 days post-seeding. A significant increase in stiffness is observed in the Fibrotic scaffold at 15 days (data are presented as mean±S.D. n = 3. Statistical significance was determined using an unpaired two-tailed Student's t-test. $*p<0.05$).

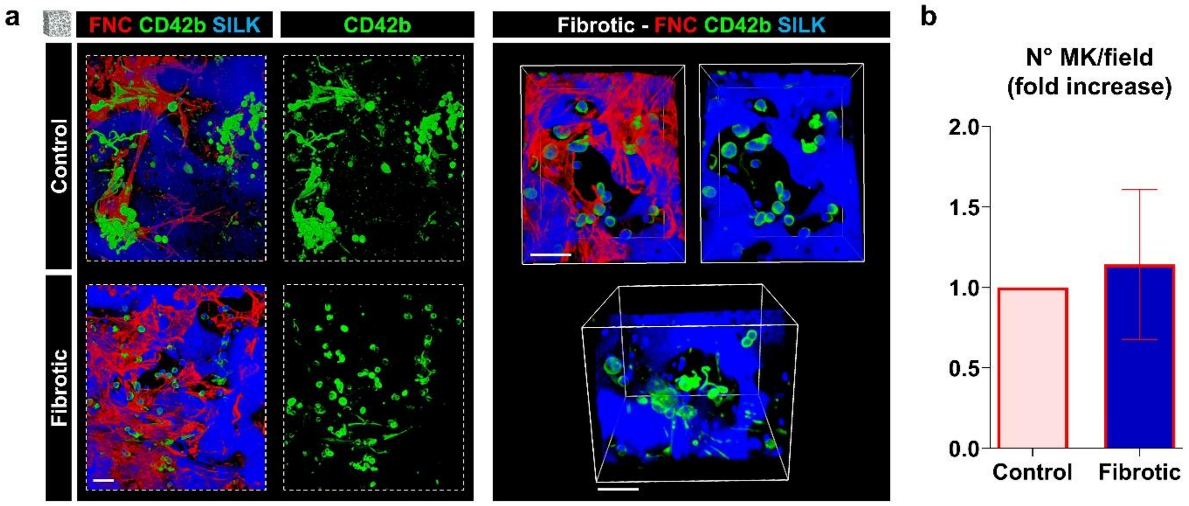

Figure S11. (a) Representative confocal images showing CD42b$^+$ megakaryocytes (green) embedded within the FIBRONECTIN (FNC) matrisome (red) inside silk fibroin scaffolds (SILK, blue) under control and fibrotic conditions. Fibrosis was induced by TGF-β1 functionalization. 3D reconstructions illustrate homogeneous spatial cell distribution within the fibrotic scaffolds (scale bars = 50 μm). **(b)** Quantification of the number of megakaryocytes per field, expressed as fold increase in the fibrotic scaffolds relative to control scaffolds. Data are shown as mean ± S.D. (n = 25 fields per condition). Statistical significance was determined using an unpaired two-tailed Student's t-test (p = NS).

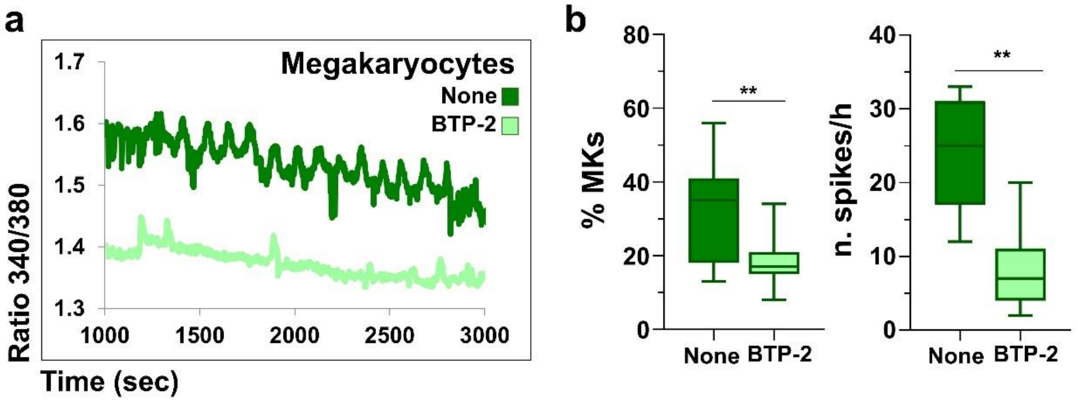

Figure S12. (a) Representative calcium (Ca^{2+}) imaging traces of mature megakaryocytes (MKs) in physiological calcium concentration (1.5 mM Ca^{2+}), in the absence (*None*, dark green) or in the presence of BTP-2 (light green). Cells in 1.5 mM Ca^{2+} display sustained intracellular Ca^{2+} spikes, whereas BTP-2 induced a marked reduction in spiking activity. **(b)** Quantification of the percentage of MKs exhibiting Ca^{2+} spikes (left) and the number of spikes per hour (right). Significant reduction in both parameters is observed under treatment with BTP-2. Data shown as box plots: boxes represent the interquartile range, horizontal lines indicate the median, and whiskers represent min/max values. Statistical significance was determined using an unpaired two-tailed Student's t-test (**$p < 0.01$).

Table S1. Parameters of force map fitting.

SF scaffold	x_c (Pa)	w (Pa)	A (normalized)
S_SF10	$2.3*10^5 \pm 3.4*10^4$	$2.7*10^5 \pm 1.8*10^4$	1
S_SF30	$1.7*10^5 \pm 4.3*10^3$	$3.2*10^5 \pm 2.1*10$	1
S_SF50	$3.7*10^4 \pm 4.0*10^3$	$7.0*10^4 \pm 8.0*10^3$	1

[a] Gaussian model of the type $y = y_0 + \frac{2A}{\pi}\frac{w}{4(x-x_c)^2+w^2}$, where y_0 is the offset, A is the area under the curve, x_c is the center of the curve and w is the width.

[b] All data are averages ± st.dev over 3 independent samples.

Table S2. Key reagents and resources.

**Reagent or resource**	**Source**	**Identifier**
Chemical compounds and drugs		
Sodium Carbonate (Na_2CO_3)	Sigma-Aldrich	#451614
Lithium Bromide (LiBr)	Sigma-Aldrich	#213225
Penicillin – Streptomycin 100X	Euroclone	#EB3001D
Paraformaldehyde (PFA)	Sigma-Aldrich	#158127
Triton X-100	Sigma-Aldrich	#X100
L-Glutamine 100X	Euroclone	#ECB300D
Non-essential amino acids 100X	Euroclone	#ECB3054D
Insulin	Sigma-Aldrich	#I9278
Dexamethasone	Calbiochem	#CAS 50-02-2
Ascorbic Acid	Sigma-Aldrich	#A4544
B-Glycerophosphate	Sigma-Aldrich	#G9891
3-Isobutyl-1-methylxanthine (IBMX)	Calbiochem	#CAS 28822-58-4
Indomethacin	Calbiochem	#CAS 53-86-1
Human Plasma Fibronectin	EMD Millipore	#FC010
Dulbecco's Phosphate Buffered Saline (PBS)	Euroclone	#ECB4053L
ProLong Gold antifade reagent	Invitrogen	#P36980
StemSpan SFEM Medium	STEMCELL Technologies Inc.	#09650
Lympholyte® H, Cell Separation Media Liquid.	Cedarlane Lab	#DVCL5026
Dulbecco's Modified Eagle's Medium High Glucose with sodium pyruvate, without L-Glutamine (DMEM)	Euroclone	#ECB7501L
MesenCult Human Supplement	STEMCELL Technologies Inc.	#5402

Hoechst 33258	Sigma-Aldrich	#861405
Peptides and recombinant proteins		
Recombinant Human Thrombopoietin (TPO)	Peprotech	#300–18
Recombinant Human interleukin-11 (IL-11)	Peprotech	#200–11
Human TGF - beta1 (HEK293 derived)	Peprotech	#100-21
Antibodies		
FITC anti-human CD61 Antibody (Mouse Monoclonal)	Beckman Coulter	#IM1758
PE anti-human CD42b Antibody (Mouse Monoclonal)	Beckman Coulter	#IM1417U
APC anti-human CD41 Antibody (Mouse Monoclonal)	Beckman Coulter	#B16894
Anti-CD61 (Mouse monoclonal)	Beckman Coulter	#IM0540
Anti-CD42b (Rabbit Polyclonal)	Invitrogen	#PA5109282
Anti-CD34 antibody [EP373Y] (Rabbit Monoclonal)	Abcam	#AB81289
FITC anti-mouse Cd41 Antibody [MWReg30] (Rat Monoclonal)	eBioscience	#11-0411-82
β1-Tubulin (Rabbit monoclonal)	Abcam	# AB179511
Anti-Osteopontin (Rabbit polyclonal)	Abcam	#AB8448
Anti-Perilipin-1 (Rabbit monoclonal)	Cell Signaling Technology	#9349
Anti-Perilipin 2 (Mouse monoclonal)	Progen	#690102S
Anti-Collagen III antibody (Rabbit polyclonal)	Abcam	#AB7778

Anti-Collagen IV antibody (Rabbit polyclonal)	Abcam	#AB6586
APC-A750 anti-human CD90 Antibody (Mouse Monoclonal)	Beckman Coulter	#B36121
PC7 anti-human CD105 Antibody (Mouse Monoclonal)	Beckman Coulter	#B43293
Anti-Fibronectin (Rabbit polyclonal)	Merck Millipore	#AB1954
Anti-Fibronectin (Mouse monoclonal)	Proteintech	#66042-1-Ig
Anti-Collagen I antibody (Rabbit monoclonal)	Abcam	#AB138492
Anti-Fibronectin antibody [IST-9] - BSA and Azide free (Mouse monoclonal)	Abcam	#AB6328
Anti-Fibronectin antibody [IST-9] (Mouse monoclonal)	Santa Cruz Biotechnology	#SC-59826
Anti-Chondroitin Sulfate antibody [CS-56] (Mouse monoclonal)	Abcam	#AB11570
Anti-α-Actin (Mouse monoclonal)	Sigma - Aldrich	#113200
Anti-α-Smooth Muscle Actin (ACTA2) antibody [1A4] (Mouse monoclonal)	Sigma - Aldrich	#A2547
Goat anti-Rabbit IgG (H+L) Secondary Antibody, Alexa Fluor 633	Invitrogen	#A-21070
Goat anti-Rabbit IgG (H+L) Secondary Antibody Alexa Fluor 488	Invitrogen	#A-11034

Goat anti-Mouse IgG (H+L) Secondary Antibody Alexa Fluor 488	Invitrogen	#A-11029
Goat anti-Mouse IgG (H+L) Secondary Antibody, Alexa Fluor 546	Invitrogen	#A-11030
Biological samples		
Human Mesenchymal Stem Cells	Lonza	#LOPT2501
Commercial Kits		
Slide-A-Lyzer Dyalisis Cassettes, 3.5K MWCO, 12 mL	Thermo Scientific	#66110
SsoFast Evagreen Supermix	Bio-Rad	#1725201
iScriptTM cDNA Synthesis Kit	Bio-Rad	#1708891
GenElute^{TM} Mammalian Total RNA Miniprep Kit	Sigma-Aldrich	#RTN70
MiniMACS Starting Kit	Miltenyi Biotec	# 130-090-312
TruCount	Becton Dickinson	#340334
Human TGF-beta1 ELISA Kit	Sigma-Aldrich	#RAB0460-1KT
Reticulum II Staining Kit	Roche	#05279399001
